# Differential diagnosis of autism, attachment disorders, complex post‐traumatic stress disorder and emotionally unstable personality disorder: A Delphi study

**DOI:** 10.1111/bjop.12731

**Published:** 2024-09-20

**Authors:** Rachel Sarr, Debbie Spain, Alice M. G. Quinton, Francesca Happé, Chris R. Brewin, Jonathan Radcliffe, Sally Jowett, Sarah Miles, Rafael A. González, Idit Albert, Alix Scholwin, Marguerite Stirling, Sarah Markham, Sally Strange, Freya Rumball

**Affiliations:** ^1^ Institute of Psychiatry, Psychology and Neuroscience King's College London London UK; ^2^ Clinical Educational & Health Psychology University College London London UK; ^3^ South London and Maudsley NHS Trust London UK; ^4^ NHS Education for Scotland Glasgow UK; ^5^ East London NHS Foundation Trust London UK; ^6^ Centre for Psychiatry Imperial College London London UK; ^7^ West London NHS Trust, London, UK; ^8^ Department of Biostatistics & Health Informatics King's College London London UK; ^9^ Oxleas NHS Foundation Trust,Dartford, UK

**Keywords:** assessment, attachment disorder, autism, borderline personality disorder, complex post‐traumatic stress disorder, diagnosis, emotionally unstable personality disorder, trauma

## Abstract

Individuals diagnosed with autism, attachment disorders, emotionally unstable personality disorder (EUPD) or complex post‐traumatic stress disorder (CPTSD) can present with similar features. This renders differential and accurate diagnosis of these conditions difficult, leading to diagnostic overshadowing and misdiagnosis. The purpose of this study was to explore professionals' perspectives on the differential diagnosis of autism, attachment disorders and CPTSD in young people; and of autism, CPTSD and EUPD in adults. A co‐produced three‐round Delphi study gathered information through a series of questionnaires from 106 international professionals with expertise in assessing and/or diagnosing at least one of these conditions. To provide specialist guidance and data triangulation, working groups of experts by experience, clinicians and researchers were consulted. Delphi statements were considered to have reached consensus if at least 80% of participants were in agreement. Two hundred and seventy‐five Delphi statements reached consensus. Overlapping and differentiating features, methods of assessment, difficulties encountered during differential diagnosis and suggestions for improvements were identified. The findings highlight current practices for differential diagnosis of autism, attachment disorders, CPTSD and EUPD in young people and adults. Areas for future research, clinical and service provision implications, were also identified.

## BACKGROUND

Differential diagnosis requires a decision to be made as to whether the criteria for a condition are met, or an alternative diagnosis could better account for the presentation. This also involves identifying possible co‐occurring conditions. Misdiagnosis occurs when an incorrect diagnosis is given, and diagnostic overshadowing refers to features of a condition being misattributed to another condition already diagnosed. Either diagnostic error can negatively impact an individual's quality of life due to inadequate care, increased psychological distress, confusion, and a loss of confidence in professionals (O'Connor & McNicholas, 2020). To increase diagnostic accuracy, it is crucial to conduct a comprehensive assessment, considering current difficulties, developmental history, differential diagnosis, and identifying therapeutic needs (Duvall et al., 2022).

Several factors can affect professionals' decision‐making during differential diagnosis. First, mental health and neurodevelopmental conditions may present similarly due to their shared features, making it challenging to identify the underlying diagnoses. For instance, overlaps between mental health conditions and autism spectrum disorder (henceforth referred to as *autism*) may result in a delayed diagnosis of autism due to overlooked autism features, insufficient awareness of autism presentations and misdiagnosis as a mental health condition (Kentrou et al., 2021). Second, professionals' decision‐making can be influenced by a range of cognitive biases, as outlined by Mouchabac et al. (2021) and Webster et al. (2021). Biases can be implicit and automatic resulting in a lack of rational consideration of all diagnostic possibilities (e.g., availability bias, confirmation bias), or explicit, affecting attitudes, beliefs, and knowledge (e.g., overconfidence bias). Additionally, it is a common practice for professionals to specialize in a specific clinical population, condition or age group, often influenced by their personal interest, professional training and theoretical framework (Aboraya et al., 2006). Such specialisms may exacerbate a tendency towards availability bias (features are interpreted through a familiar lens), confirmation bias (information prioritized confirms current thinking), or overconfidence bias (professionals believe they know more than they do) (Mouchabac et al., 2021; Webster et al., 2021). Professionals may also have a bias towards diagnosing a condition perceived as less stigmatizing or preferred by the individual being assessed (Atkinson et al., 2024). Finally, service and individual factors such as workload or fatigue can further predispose professionals to cognitive biases which can impact diagnostic decisions (Webster et al., 2021).

Individuals diagnosed with autism, attachment disorders, complex post‐traumatic stress disorder (CPTSD) and emotionally unstable personality disorder (EUPD) may present with overlapping features that can similarly impact emotional, behavioural and interpersonal functioning (Gordon & Lewis, 2020; Karatzias et al., 2023; Ng‐Cordell et al., 2022; Zeanah et al., 2016). While overlaps exist between many diagnoses, the decision to focus on these four conditions was based on the authors' professional experiences and a need identified in clinical practice. Attachment disorders are typically diagnosed in young people, while EUPD is more often diagnosed in adults. While autism and CPTSD can be diagnosed in both age groups, the former is a neurodevelopmental condition, whereas trauma is the cause of the latter. Given the substantial overlaps between these conditions, it is unsurprising that misdiagnosis and diagnostic overshadowing are commonly reported (Au‐Yeung et al., 2019; Porr, 2017; Woolgar & Scott, 2014). However, accurate diagnosis is often necessary to receive appropriate support (Jablensky, 2016). Autism misdiagnosed as a mental health condition may result in unnecessary treatment (e.g., medication) and unmet needs (Au‐Yeung et al., 2019). Similarly, with trauma or adversity‐related difficulties, misdiagnosis reduces the quality of life and access to evidence‐based treatment (Elliott et al., 2021). On the other hand, misdiagnosis of EUPD can lead to stigma, mistreatment and negatively impact quality of life (Porr, 2017).

Although characterized by varying phenomenology, autism is defined by difficulties in social communication and interactions, and restricted interests and repetitive behaviours (World Health Organization, 2019). Similarly, difficulties in interactions are characteristic of attachment disorders, but these result from inadequate caregiving environments before the age of 5 (Zeanah & Gleason, 2015). Two clinical forms of attachment disorders have been defined within diagnostic manuals (World Health Organization, 2019): inhibited or withdrawn, known as reactive attachment disorder (RAD), and disinhibited or social, known as disinhibited social engagement disorder (DSED). Although ‘attachment disorder’ is often used by professionals to describe children with complex presentations who have had difficult early life experiences, diagnosed attachment disorders are rare (Woolgar & Scott, 2014). Practice parameters have indicated that children with RAD or DSED and autistic children have overlapping features with regard to social communication and motor stereotypies, but can be differentiated by autism‐related restricted interests and the quality of social difficulties (Zeanah et al., 2016).

Like attachment disorders, International Classification of Diseases (ICD)‐11 CPTSD^1^ can be linked to caregiving relationships with trauma of an interpersonal nature (e.g., abuse, neglect) (Hyland et al., 2021; Sarr et al., 2024). CPTSD typically develops following exposure to prolonged and/or repetitive event(s) of a threatening or horrific nature. It is defined by the core features of post‐traumatic stress disorder (PTSD) (re‐experiencing, avoidance and sense of threat) and disturbances in self‐organization (affect dysregulation, negative self‐concept and difficulties in relationships) (World Health Organization, 2019). Similarly, EUPD^2^ is characterized by difficulties in emotional regulation and relationships (World Health Organization, 2016). Individuals with CPTSD or EUPD overlap substantially in terms of a negative perception of self, relational and affect dysregulation (Karatzias et al., 2023). Overlaps also exist between EUPD and autism in terms of difficulties with communication, emotional and social functioning (Gordon & Lewis, 2020). Differences include difficulties in the use of non‐verbal communication, narrow interests, insistence on sameness and repetition specific to autism, but not EUPD (Lai & Baron‐Cohen, 2015).

Accurate diagnosis of autism, attachment disorders, CPTSD and EUPD is further affected by the impact of early trauma and adverse life events, co‐occurring conditions, and a heavy reliance on retrospective reporting resulting in difficulties assessing aetiology and trajectory of symptoms (Fusar‐Poli et al., 2022; Morgan & Zimmerman, 2014). While trauma is a prerequisite for CPTSD diagnosis, a trauma history is also reported by a high proportion of individuals diagnosed with EUPD (30%–90%, Bozzatello et al., 2021) and autism (95%, Rumball et al., 2021). Although trauma history rates in attachment disorders are not documented, trauma such as neglect, maltreatment or abuse are often intrinsic to attachment disorders. A qualitative analysis of interviews with professionals has revealed that professionals use the term “attachment disorders” interchangeably with trauma (Coughlan et al., 2022). Not all individuals who experience early trauma or adversity develop mental health difficulties. However, compared to individuals with mental health difficulties who have not faced these experiences, those who do tend to present with more complex presentations, co‐occurring conditions, and functional impairments which may be harder to differentiate diagnostically (McKenzie & Dallos, 2017). For example, signs of trauma such as social withdrawal and emotional avoidance can be mistaken for autism‐related behaviours (Ng‐Cordell et al., 2022).

Misdiagnoses can lead to missed opportunities for interventions (Ng‐Cordell et al., 2022). As a result, efforts have been made to develop tools to assist with differential diagnosis. For instance, The Coventry Grid (Moran, 2010) and Coventry Grid Interview (Flackhill et al., 2017) are designed to aid in differentiating autism and attachment problems. However, these tools are grounded in the authors' clinical practice and do not map onto attachment disorders as defined by the ICD‐11 as they are based on attachment theory, which has been suggested to have limited psychopathological significance (Rutter et al., 2009). Similarly, published guidelines for differentiating between autism and EUPD (Gordon & Lewis, 2020) also rely on the authors' clinical experience and a synthesis of the literature. Recently, the World Health Organization (2024) published guidance to aid in distinguishing between ICD‐11 conditions, but information is not provided to distinguish CPTSD from autism and attachment disorders.

Past research and guidelines have explored the similarities and differences between these conditions as dichotomies (i.e., autism and attachment disorders, autism and EUPD, CPTSD and EUPD). Although this is a useful starting point, given the overlap in symptomatology and impairment, there is a need to study differential diagnosis across these conditions simultaneously and understand professionals' obstacles when attempting to diagnostically differentiate between these conditions. This study used a Delphi method to identify expert professionals' views about best practice in differential diagnosis of autism (without intellectual disability), CPTSD and attachment disorders in young people aged 7 to 17, and autism (without intellectual disability), CPTSD, and EUPD in adults aged 18 years and above. Due to the already broad scope, this study did not set out to explore the co‐occurring presentations of the conditions or to investigate the underlying psychological mechanisms and associations between the conditions.

## METHODS

### Study design

A co‐produced three‐round Delphi methodology was used to gather information from experts through a series of questionnaires. The Delphi technique is utilized to develop consensus on complex issues through an iterative design (Hsu & Sandford, 2007). This methodology has previously been used for the development of clinical recommendations in the absence of larger‐scale research or published guidance (Jünger et al., 2017). The framework was based on the models described by Hasson et al. (2000), Hsu and Sandford (2007), and Jünger et al. (2017). Delphi studies have a minimum of 10 to 15 participants in the first round, relying on the quality of the expertise of participants over the quantity (Hsu & Sandford, 2007).

Two specialist working groups informed the study conceptualization, design and interpretation. The first group consisted of five individuals with lived experience of receiving at least one of the diagnoses explored. The second group consisted of eight professionals with specialist clinical and/or research expertise in autism, attachment disorders, CPTSD and EUPD. Decisions made during the study were based on the majority opinions of the working groups.

Ethical approvals were obtained from King's College London (MRSP‐22/23–34,011).

### Participants

A combination of purposive, convenience and snowballing sampling was used, through the authors' professional networks, word of mouth, social media, and professionals' organizations. Participants were provided with information sheets and gave written informed consent to take part in the study.

Participants were professionals worldwide with expertise in the assessment and/or diagnosis of at least one of the explored conditions. The inclusion criteria were:

- Having a relevant core clinical profession with a minimum of 4 years of experience post‐qualification.
- Experience assessing and/or diagnosing at least one of the explored conditions.
- Using English proficiently to complete the survey.^3^

**FIGURE 1:**
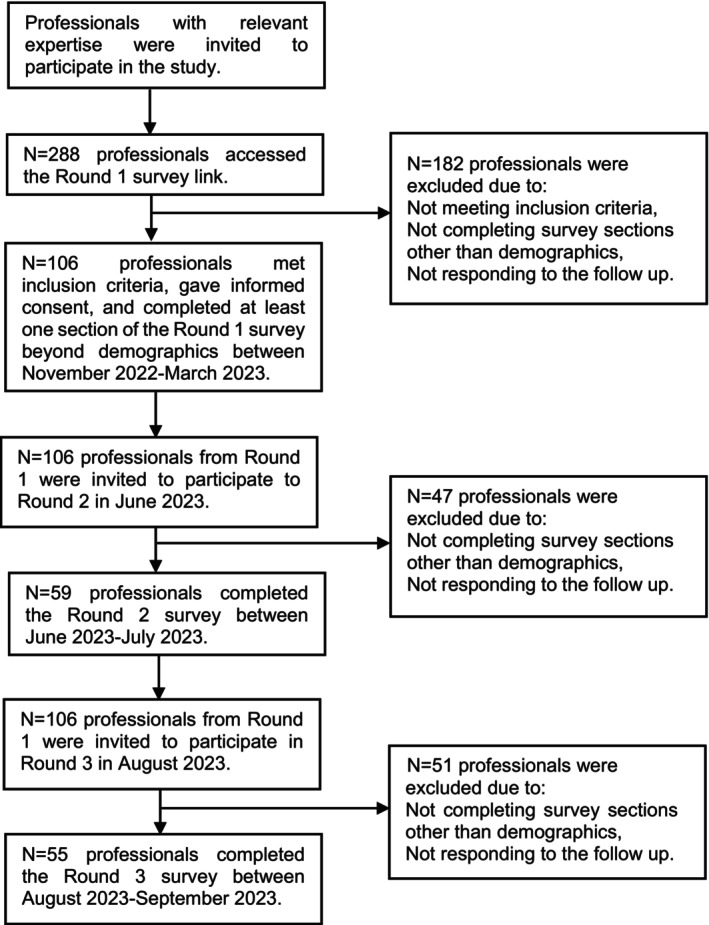
Flowchart of the enrolment and follow‐up of participants.

A total of 106 participants took part in the Round 1 survey (Figure 1). Of these, 59 participants took part in Round 2 (56% retention rate). In Round 3, 55 participants took part (93% retention rate from Round 2; 52% retention rate across all rounds). Participation in Round 1 was required for Round 2 and 3, but participation in Round 2 was not a requirement for Round 3. Of the included participants, there were 12 incomplete surveys in Round 1, three in Round 2 and one in Round 3. Surveys were included if at least one section was completed beyond demographics. Participants' demographics can be found in Table 1, with further information in Table S1.

**TABLE 1:** Characteristics of participants.

	Round 1 (*n* = 106)	Round 2 (*n* = 59)	Round 3 (*n* = 55)
**Profession**			
Clinical psychologist	57	33	35
Counselling psychologist	3	2	1
Educational psychologist	7	6	4
Forensic psychologist	3	0	0
Neurodevelopmental practitioner	1	1	1
Occupational therapist	3	1	1
Psychiatrist	16	10	8
Psychotherapist	5	1	1
Registered nurse	1	1	1
Social worker	3	2	2
Speech and language therapist	7	2	1
**Country of work**			
Australia	8	6	6
Canada	5	4	2
Georgia	1	1	1
Germany	1	1	1
Hong Kong	1	1	1
Ireland	4	3	1
Switzerland	1	1	1
United Arab Emirates	1	0	0
United Kingdom	70	36	38
United States of America	14	6	4
**Work setting**			
Adults' mental health service in the community	23	10	10
Children and adolescents' mental health service in the community	27	20	17
Forensic service	13	4	7
Inpatient mental health service	6	3	2
Independent/private practice	41	22	18
School	6	5	2
Specialist service	41	21	24
University	14	7	6
Voluntary sector organization	2	0	1
Not currently working clinically	1	0	0
**Main age group worked with**			
Children and/or adolescents	50	23	27
Adults	56	36	28
**Years of clinical experience** ^a^			
4–9 years	42	27	21
10–14 years	24	12	13
15 years or more	40	20	21
**Areas of expertise** ^a^			
Attachment disorders	17	13	8
Autism	70	42	36
CPTSD	51	27	27
EUPD	26	11	11
Others	26	12	10
**Main area of expertise**			
Attachment disorders	7	2	4
Autism	58	33	28
CPTSD	30	18	17
EUPD	11	6	6
**Years of experience worked with expertise condition** ^a^			
Less than 4 years	6	0	2
4–9 years	44	27	23
10–14 years	21	12	11
15 years or more	35	20	19
**Number of assessments and/or diagnoses of expertise condition** ^a^			
1–9	6	5	5
10–24	10	5	4
25–49	18	11	10
50 or more	72	38	36
**Current frequency of assessing and/or diagnosing expertise condition** ^a^			
Occasionally (every couple of months or less)	21	14	13
Sometimes (at least once a month)	30	14	14
Regularly (at least weekly)	54	31	28
Did not answer	1	0	0

^a^ Questions asked at Round 1 only.

**FIGURE 2:**
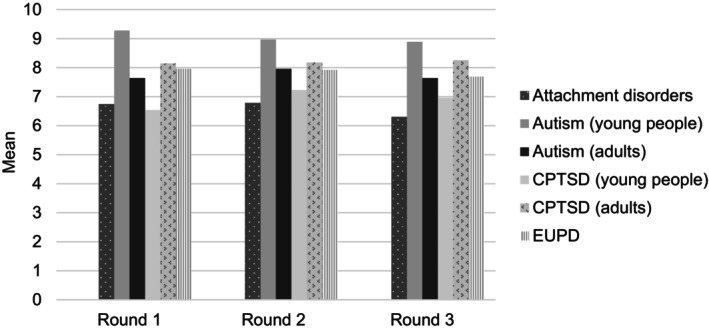
Participants' self‐rated knowledge and understanding of the core diagnostic features of expertise condition in each age group (young people and adults). Data are means of ratings from 1 (know very little) to 10 (very well informed). Question asked at Round 1 only.

Most of the participants were psychologists and psychiatrists employed in Western countries. Participants were asked to indicate the condition they primarily worked with, and autism experts had the highest representation, followed by CPTSD experts. The number of experts in attachment disorders and EUPD was low. This was reflected in the lower knowledge and understanding of attachment disorders reported by participants across all rounds (Figure 2). Most participants had conducted 50 or more diagnostic assessments and did them regularly. The majority of the participants had experience working with more than one of the explored conditions. The lowest knowledge and understanding of co‐occurring conditions and differential diagnosis was for CPTSD in young people (Table S1).

### Procedure

Questionnaires were created on Qualtrics. Only relevant questions were displayed to participants, based on their clinical expertise and the age group they worked with. The surveys were piloted by the research team and external parties. To link demographic data and responses across the Delphi rounds, the participants were assigned an anonymous participant identifier at Round 1. In between rounds, participants were sent a summary of the previous rounds including participant demographics, previous round findings and next round instructions. The rounds took place from November 2022 to September 2023.

**Round 1 survey**. An online semi‐structured questionnaire was built based on existing research and input from working groups. This focused on: (1) professional background and basic demographics; (2) features suggestive of autism or attachment disorders or CPTSD or EUPD; and (3) factors to consider in the assessment, diagnosis and differential diagnosis of these conditions. Although the consent form served as an initial screening tool to ensure participants met inclusion criteria, further questions in the demographics section screened eligibility.

**Round 2 survey**. The findings from the first survey and feedback from working groups were used to create the Delphi statements. As a result, the Round 2 survey focused on: (1) overlapping and differentiating features; (2) co‐occurrence considerations; (3) assessment methods for differential diagnosis; and (4) difficulties encountered during differential diagnosis and suggestions for improvements. Participants were asked to rate their agreement with each Delphi statement on a 5‐point Likert scale with the additional options of selecting “I don't know” or “I agree with part of this statement”. They could also provide additional information through written responses.

**Round 3 survey**. Findings from the second survey and input from working groups were used to create the final survey. This included new statements issued from the Round 2 text responses. Several statements previously rated during Round 2 were included for re‐rating, along with their original agreement percentage. The Round 3 survey covered the same categories as the Round 2 survey. Participants were asked to rate the statements using the same scale and were given the option to provide additional text information.

### Data analysis

Data were analysed using mixed methods. Qualitative data issued from participants' text responses were analysed using content analysis. This approach is widely used to analyse open‐ended questions in surveys and Delphi studies (Hasson et al., 2000). The content analysis method described by Stemler (2000) was used with NVivo software. The steps were as follows: (1) defining categories based on the research aims and areas explored at each round; (2) a priori coding (also called deductive content analysis) where text was coded to these categories; (3) emergent coding (or inductive content analysis) where subcategories were established. These subcategories were used as the basis for the Delphi statements. While the content of the statements remained largely unchanged, working groups helped to clarify and/or improve them. The triangulation of the data, with the added confirmation of findings and alternative perspectives from working groups, contributed to the validity (Carter et al., 2014).

Descriptive statistics on Microsoft Excel were used for the quantitative data analysis. This calculated the mean level of agreement among respondents. Consensus was considered to have been reached when at least 80% of the participants agreed with a Round 2 or Round 3 statement (Hsu & Sandford, 2007). Round 2 statements that received 60 to 79% agreement were re‐rated at Round 3 and those with less than 60% agreement were excluded. Due to the study being limited to three rounds, Round 3 statements with agreement levels below 80% agreement were excluded.

## RESULTS

Overall, 275 statements were endorsed by participants. The results are summarized in Table 2. The full list of statements is found in Table 3. The number of participants who contributed to each category is reported in Table S2, and further information regarding the number of statements during each round is found in Figure S1. Statements that were not endorsed by participants are found in Table S3.

**TABLE 2:** Similarities and differences between all conditions.

Features	Autism		CPTSD		Attachment disorders	EUPD
	Young people	Adults	Young people	Adults		
Family history of neurodevelopmental conditions	X	X				
History of insufficient care by age 5					X	
History of adverse experiences			X	X	X	X
Presents from an early age	X	X			X	
Presents in adolescence or young adulthood						X
Presents following traumatic events			X	X		
Difficulties with insight into own emotions	X	X	X	X	X	X
Co‐occurring anxiety	X	X	X	X	X	X
Co‐occurring low mood		X		X		X
Mood is typically stable and negative				X		
Mood is unstable						X
Fears of coming to harm again				X		
Varied fears (e.g., rejection, abandonment)						X
Difficulties regulating emotions	X	X	X	X	X	X
Difficulties with sense of identity and/or self‐esteem	X	X		X	X	X
Feeling different from others	X		X			
Sense of not belonging and misunderstood		X				X
Difficulties with mentalization		X				X
Difficulties coping with uncertainty and/or need for predictability		X		X		X
Rigidity	X	X	X	X	X	X
Impulsivity				X		X
Risk				X		X
Difficulties with cognitive functioning	X	X	X	X		
Sleep difficulties	X	X	X	X		
Food difficulties	X				X	
Dissociative experiences				X		X
Re‐experiencing symptoms			X	X		
Repetitive and stereotyped behaviours or movements	X	X				
Repetitive play	X		X			
Special interests	X	X				
Sensory processing difficulties	X	X	X	X	X	X
Sensory sensitivities are trauma‐related			X	X		
Sensory sensitivities revolve around day‐to‐day	X	X				
Difficulties forming and maintaining relationships and in other interpersonal aspects	X	X	X	X	X	X
Social difficulties due to inherent difficulties in social communication, social skills and understanding	X	X				
Social difficulties due to trust difficulties and/or lack of safety			X	X		
Social difficulties due to patterns of extremely negative experiences with caregivers					X	
Social difficulties due to idealization or devaluation of self and others						X
Hypervigilance to subtle signs signalling disapproval and/or mistrust						X

X indicates that the feature is associated with the condition and age group. This is a summary of features based on the Delphi statements that reached consensus (Table 3).

**TABLE 3:** Consensus statements.

Statements relating to young people	Participants agreement (%)
**Overlapping features in autism and CPTSD**	
Co‐occurring anxiety (e.g., preoccupations, rumination).	88
Difficulties identifying/labelling own emotions and processing/understanding own emotions. *This can be due to underlying alexithymia in autism and the resulting impact of trauma in CPTSD*. *(P)*	100
Difficulties regulating emotions (e.g., flattened affect, outbursts, or a fluctuation between the two). *Emotions can feel intense and uncontrollable in CPTSD*. *(WG2)*	97
Cognitive rigidity (e.g., thoughts) and behavioural rigidity (e.g., preference for routine and predictability). *Rigidity can present as pervasive and global negative self‐concept in CPTSD. (WG2) Avoiding change can result in rigidity in CPTSD*. *(P)*	83
Difficulties with cognitive functioning (e.g., concentration, executive function).	83
Sleep disturbances.	88
Sensitivity to specific sensory stimuli and environments (e.g., startle reaction, distress).	82
Difficulties expressing emotions to others.	92
Difficulties understanding and interpreting others' emotions and intentions. *This can present as relationship avoidance and/or feeling cut off from others in CPTSD*. *(WG2)*	92
Difficulties forming and maintaining relationships.	92
Feeling different from others.	91
**Differentiating features between autism and CPTSD**	
Difficulties are evident from birth or an early age in autism, but they occur following traumatic events in CPTSD. *Autism can first show signs from the ages of 6 to 12 months*. *(WG2)*	88
Autism is characterized as lifelong, whereas the lifelong nature of CPTSD can vary based on its impact on the developing brain, the developmental timing, the nature of the trauma and response to evidence‐based interventions. *Learned behaviours resulting from early repetitive interpersonal trauma can be difficult to change*. *(WG1)*	96
There is typically a family history of neurodevelopmental conditions in autism, whereas there may be family reports of traumatic events in CPTSD. *There is often a family history of neurodevelopmental conditions in autism*. *(WG2)*	96
Mental health difficulties may be absent or present in autism, whereas they are by definition always present in CPTSD.	91
Re‐experiencing symptoms (e.g., flashbacks, intrusive thoughts, nightmares) are not characteristic of autism, whereas they are indicative of CPTSD.	91
Intrusive memories or flashbacks can relate to atypical traumatic events (e.g., social mistakes) in autism, whereas intrusive memories or flashbacks are generally related to typical traumatic events in CPTSD. *The traumatic nature of an event may be based on subjective threat perception in autism*. *(WG2)*	88
Repetitive behaviours or movements (e.g., stimming, play) can be characteristic of autism, whereas repetitive play is due to the processing of traumatic events in CPTSD. *Repetitive play in CPTSD typically occurs in younger children*. *(WG2) Repetitive play in CPTSD can be an emotion regulation mechanism*. *(P)*	92
Special interests that are unusual in nature and intensity are indicative of autism, whereas they are not characteristic of CPTSD.	82
Sensory sensitivities revolve around day‐to‐day stimuli in autism, whereas they are trauma‐related in CPTSD. *There can be triggers related to sensory overload in autism, but also social stressors and engaging in non‐preferred activities. (P) Sensitivity and triggers in CPTSD may not be obviously related to the trauma*. *(P)*	92
Language and social communication difficulties (e.g., idiosyncratic or repetitive language, reciprocity) are indicative of autism, whereas they are not characteristic of CPTSD. *Atypical language and/or language difficulties are common in autism but typically occur in the context of co‐occurring speech delay, intellectual disability, or learning disability*. *(WG2)*	96
Social communication difficulties are due to inherent differences associated with neurodiversity (e.g., central coherence, theory of mind, executive functioning) in autism, whereas they are due to difficulties related to the impact of trauma (that may cause neurobiological changes) in CPTSD. *Social communication difficulties in CPTSD can be due to executive functioning struggles*. *(P)*	88
Social difficulties are due to neurodevelopmental differences or impairments in autism, whereas they are due to trust difficulties or a lack of interpersonal safety in CPTSD.	85
Relationship difficulties are due to difficulties in perspective‐taking or social problem‐solving in autism, whereas they are mainly due to distrust of others and fear of being harmed or emotional regulation difficulties or negative self‐concept in CPTSD. *Many autistic individuals can be quite content with “limited” social contact. The desire for ‘more friends' can be prevalent in pubescent‐aged females or rather than being a part of a big social group, they tend to have quite intense small groups of friends. (P) Relationship difficulties can also be due to trauma reminders in CPTSD*. *(WG2)*	92
**Overlapping features in autism and attachment disorders**	
Co‐occurring anxiety (e.g., around new places, new people).	92
Difficulties identifying/labelling own emotions and processing/understanding own emotions.	80
Difficulties regulating emotions (e.g., flattened affect, outbursts, or a fluctuation between the two).	88
Difficulties with sense of identity and self‐esteem.	83
Cognitive rigidity (e.g., rules, views) and behavioural rigidity (e.g., preference for routine and control).	86
Difficulties related to food (e.g., selectivity, hoarding). *Underlying difficulties may be related to sensory processing*. *(WG2)*	81
Sensory processing difficulties (e.g., aversion or sensory seeking).	88
Difficulties with non‐verbal communication (e.g., eye contact, facial expressions).	86
Difficulties forming and maintaining relationships.	92
Difficulties understanding and interpreting others' emotions and intentions.	88
Difficulties initiating social overtures (e.g., seeking comfort, asking for help).	88
**Differentiating features between autism and attachment disorders**	
Aetiology is neurodevelopmental in autism, whereas aetiology is the nature of care from caregivers in attachment disorders. *Attachment disorders aetiology is neglect or multiple caregivers (who may well be all adequate/positive, but without enduring relationships) or institutionalization with high child‐caregiver ratios*. *(P)*	80
A functioning attachment system does not impact autism diagnosis, whereas it impacts attachment disorders diagnosis. *While attachment disorders can persist, with consistent and supportive care, attachment disorders can also be resolved*. *(P)*	80
Repetitive and stereotyped behaviours or movements (e.g., stimming) are characteristic of autism, whereas they are not characteristic of attachment disorders. *Due to limited development opportunities, behaviours may appear repetitive in attachment disorders*. *(WG2)*	91
Special interests that are unusual in nature and intensity are characteristic of autism, whereas they are not in attachment disorders.	80
Sharing of enjoyment is reduced in autism, whereas seeking social approval and attention is common in disinhibited social engagement disorder. *Seeking approval and attention is secondary to primary social disinhibition in DSED*. *(P)*	82
Inherent understanding of others can be literal and concrete in autism, whereas it is not in attachment disorders. *Understanding others can be literal and concrete in attachment disorders*. *(WG2)*	82
Social disinhibition can be due to difficulties processing social information (e.g., literal understanding) or a lack of awareness of social rules in autism, whereas it is due to past negative interactions with caregivers in attachment disorders. *Social disinhibition in attachment disorders can also be due to a lack of experiences and opportunities for learning and internalized social rules*. *(WG2) There are extremely negative experiences with caregivers with attachment disorders. (P)*	91
**Overlapping features in CPTSD and attachment disorders**	
History of adverse interpersonal experiences (e.g., insufficient care or neglect, interpersonal trauma). *History of adverse interpersonal experiences Is common in CPTSD, but not necessary*. *(WG2)*	82
Difficulties regulating emotions (e.g., irritability or anger, limited positive affect).	82
Difficulties forming and maintaining relationships. *Characteristics of DSED (*e.g.*, high social disinhibition, low avoidance) are unlikely to overlap with CPTSD*. *(P)*	80
**Differentiating features between CPTSD and attachment disorders**	
A history of insufficient care (e.g., neglect, caregiver change, caregiver absence, caregiver illness or death, institutionalization) is not necessary for CPTSD, whereas it is for attachment disorders. *Neglect could be traumatizing in the sense of CPTSD, but there is not enough evidence in this area and there are different assumptions in practice*. *(P)*	82
Extremely threatening/horrific and/or prolonged/repetitive events are necessary for CPTSD, whereas they are not for attachment disorders. *Prolonged repeated trauma is common in CPTSD, but not necessary*. *(WG2)*	80
Exposure to traumatic events can occur at any age in CPTSD, whereas insufficient care must happen by age 5 for attachment disorders.	90
Re‐experiencing symptoms (e.g., flashbacks, intrusive thoughts, nightmares) are characteristic of CPTSD, whereas they are not characteristic of attachment disorders.	80
**Considerations for autism and CPTSD co‐occurrence**	
Autism is a risk factor for the development of CPTSD (e.g., social and communication difficulties can increase the risk for vulnerability or maltreatment or traumatic events). *A “difference” from a young age can increase the likelihood of abuse, victimization, which means more likelihood, to be exposed to traumatizing events*. *(P)*	82
Autism‐related difficulties can overshadow CPTSD‐related difficulties. *This can be bidirectional*. *(P)*	87
Successful intervention for CPTSD can make it easier to confirm whether an autism diagnosis is pertinent.	96
**Considerations for autism and attachment disorders co‐occurrence**	
Autistic young people can have general difficulties with attachment, without meeting attachment disorder criteria.	100
It is important to establish the trajectory of features over time (e.g., whether attachment disorders‐related difficulties are outstanding (i.e., remain) following an extended period in a stable and safe environment).	81
Autism and disinhibited social engagement disorder can co‐occur in the context of insufficient care. *Autism and attachment disorders cannot co‐occur according to diagnostic classification and existing evidence, as autism is an exclusionary criterion. If co‐occurrence seems likely, autism must be assessed first. This is not the case for DSED*. *(P)*	90
**Considerations for CPTSD and attachment disorders co‐occurrence**	
Traumatic events and/or neglect can be risk factors in young people developing CPTSD and attachment disorders.	81
CPTSD and attachment disorders are not mutually exclusive and can co‐occur in specific circumstances. *Some DSO aspects of CPTSD might co‐occur with the relationship difficulties that can accompany persistent DSED*. *(P)*	81
**Assessment methods for differential diagnosis of autism and CPTSD**	
Comprehensive history taking (clinical interview) with parents and/or caregivers. *It is essential for history taking to include family history and developmental history*. *(WG2)*	94
Comprehensive history taking (clinical interview) with young people. *It can depend on the age, developmental level and expressive communication*. *(P)*	91
Dating the onset of symptoms relative to traumatic events.	94
Referring to diagnostic criteria (e.g., DSM, ICD).	91
Autism measures (e.g., ADI, ADOS, CARS, DISCO, SCQ).	94
Traumatic event exposure and trauma symptoms measures (e.g., CAPS, CATS, CPSS, CRIES, PCL, TSCC, TSCYC).	94
CPTSD‐specific measures (e.g., ITQ‐CA).	81
Collateral information from school and/or other professionals (e.g., clinical interview, reports).	91
Observation of young people during clinical interviews or play sessions.	94
Observation of young people across two or more settings (e.g., home, school).	94
Completion of measures by parents and/or caregivers.	94
Completion of measures by school and/or other professional.	91
Discussion with a multi‐disciplinary team.	97
**Assessment methods for differential diagnosis of autism and attachment disorders**	
Comprehensive history taking (clinical interview) with parents and/or caregivers. *It is essential for history taking to include family history* (e.g.*, parental mental health, substance use), developmental history and change in placements (if applicable)*. *(WG2)*	96
Comprehensive history taking (clinical interview) with young people. *It can depend on the age, developmental level and expressive communication*. *(P)*	84
Referring to diagnostic criteria (e.g., DSM, ICD).	92
Autism measures (e.g., ADI, ADOS, CARS, DISCO, SCQ).	96
Attachment disorders measures (e.g., DAI).	80
Collateral information from school and/or other professionals (e.g., clinical interview, reports).	96
Observation of young people during clinical interviews or play sessions.	100
Observation of young people and caregivers or strangers.	88
Observation of young people across two or more settings (e.g., home, school).	100
Discussion with a multi‐disciplinary team.	96
**Assessment methods for differential diagnosis of CPTSD and attachment disorders**	
Comprehensive history taking (clinical interview) with parents and/or caregivers.	91
Comprehensive history taking (clinical interview) with young people.	82
Dating the onset of symptoms relative to traumatic events.	91
Referring to diagnostic criteria (e.g., DSM, ICD).	91
Traumatic event exposure and trauma symptoms measures (e.g., CAPS, CATS, CPSS, CRIES, PCL, TSCC, TSCYC).	91
Attachment disorders measures (e.g., DAI).	82
Collateral information from school and/or other professionals (e.g., clinical interview, reports).	82
Observation of young people and caregivers or strangers.	91
Observation of young people during clinical interviews or play sessions.	91
Observation of young people across two or more settings (e.g., home, school).	91
Discussion with a multi‐disciplinary team.	91
**Difficulties in distinguishing autism and CPTSD**	
Traumatic events occurring at a young age render it difficult to differentiate between autism and CPTSD.	81
Chronic traumatic events (i.e., persistent, repetitive) may not have a clear onset and CPTSD can be overlooked.	91
Information provided by young people and informants is not always complete (e.g., informants are unreliable, absence of developmental history, no clear onset of difficulties, language barrier). *This may also be due to shame of CPTSD symptoms and seeking support for comorbidity instead (e.g., depression)*. *(WG2)*	96
Autism is less recognized in young people who have learned to compensate for or camouflage social communication difficulties and can be overlooked.	96
Autism is less recognized and underdiagnosed in females.	81
Autism is less recognized and underdiagnosed in ethnic minoritised young people.	87
Young people under social care are assumed to have CPTSD and autism is overlooked. *Young people under social care are specifically assumed to have developmental trauma and/or complex trauma*. *(WG2)*	91
Young people who are refugees or asylum seekers are assumed to have CPTSD and autism is overlooked.	100
Cultural norms and expectations influence interactions and social communication, and the perception of traumatic events and resilience (e.g., shame, help‐seeking).	100
Access to assessment is unequal for those who experience marginalization which may cause an escalation in presentation.	84
Clinicians do not have adequate training, knowledge or confidence to assess some of the subtle aspects of autism (e.g., gender differences).	87
Clinicians do not have adequate training, knowledge or confidence to recognize trauma‐related difficulties. *There may be avoidance in diagnosing CPTSD, possibly due to it being a new concept or services not using ICD‐11*. *(WG2)*	96
**Suggestions for improved distinction between autism and CPTSD**	
Additional time to assess and/or diagnose (e.g., multiple appointments, comprehensive history and formulation, postponing diagnosis until clearer picture).	91
Involvement of the multi‐disciplinary team in all assessments.	84
Access to specialist supervision or consultation services on autism and/or CPTSD for assessing clinicians.	97
Improved training and knowledge of clinicians on autism and/or CPTSD.	97
Improved young people's CPTSD diagnostic criteria.	91
Development of guidelines and/or tools for differential diagnosis.	100
Inclusion of items exploring traumatic events in autism‐specific assessments (e.g., DISCO, ADI) or use of trauma measures in all autism assessments.	96
Further research on differential diagnosis.	100
**Difficulties in distinguishing autism and attachment disorders**	
The COVID‐19 pandemic has resulted in young people having fewer opportunities for play and interactions leading to social difficulties.	86
The COVID‐19 pandemic has resulted in fewer opportunities for observation during assessments.	90
Information provided by young people and informants is not always complete (e.g., informants are unreliable, absence of developmental history, no clear onset of difficulties, language barrier).	84
Young people under social care are assumed to have attachment disorders and autism is overlooked.	95
Autism is less recognized and underdiagnosed in females.	90
Cultural norms and expectations influence interactions, social communication and parenting style, and the perception of traumatic events and resilience.	96
Access to assessment is unequal for those who experience marginalization which may cause an escalation in presentation.	88
Clinicians do not have adequate training, knowledge or confidence on attachment disorders (e.g., reactive attachment disorder or disinhibited social engagement disorder).	83
Clinicians do not have adequate training, knowledge or confidence on the impact of early life neglect and view difficulties from a trauma lens.	81
There are insufficient sensitive and specific measures to aid clinical differentiation between autism and attachment disorders. *Most measures are not evidence‐based*. *(WG2)*	96
Differential diagnosis decisions can depend on the services accessed pre‐diagnosis and/or on the service accessed for assessment (e.g., service speciality, clinicians' clinical knowledge, nature of the assessment offered).	95
**Suggestions for improved distinction between autism and attachment disorders**	
Additional time to assess and/or diagnose (e.g., multiple appointments, comprehensive history and formulation, postponing diagnosis until clearer picture).	100
Involvement of the multi‐disciplinary team during all assessments.	92
Improved training and knowledge of clinicians on autism and attachment disorders.	96
Improved awareness on the relative low prevalence of attachment disorders.	88
Review as much collateral information as possible (e.g., chase up informants, access health visitor records, speak to professionals involved in care).	100
Development of appropriate attachment disorder assessment tools.	92
Use of attachment‐based interviews (e.g., Coventry Grid, CAI). *These tools measure attachment patterns and are not specific to diagnosable attachment disorders*. *(WG2)*	81
Inclusion of attachment disorder in autism‐specific assessments (e.g., DISCO, ADI) or use of attachment measures in all autism assessments.	81
Avoid over‐reliance on one single tool or measure.	100
Development of guidelines and/or tools for differential diagnosis.	92
Further research on differential diagnosis.	100
**Difficulties in distinguishing CPTSD and attachment disorders**	
Insufficient care occurring in the early years (e.g., neglect) can be an aetiological factor for both attachment disorders and CPTSD.	80
Extreme neglect is an aetiological factor for attachment disorders, and environments with extreme neglect may also involve abuse, an aetiological factor for CPTSD.	90
Developmental trauma can be wrongly given as a diagnosis instead of differentiating between CPTSD and attachment disorders.	80
Information provided by young people and informants is not always complete (e.g., informants may not be reliable, absence of developmental history, no clear onset of difficulties, language barrier). *This may also be due to shame of CPTSD symptoms and seeking support for comorbidity instead* (*e.g., depression)*. *(WG2)*	91
Cultural norms and expectations influence the perception of traumatic events and resilience.	91
Clinicians do not have adequate training, knowledge or confidence on attachment disorders (e.g., reactive attachment disorder or disinhibited social engagement disorder).	91
Accurate differentiation between CPTSD and attachment disorders requires detailed assessment and resources, which most services do not have available to them.	80
**Suggestions for improved distinction between CPTSD and attachment disorders**	
Additional time to assess and/or diagnose (e.g., comprehensive history).	91
Involvement of the multi‐disciplinary team in all assessments.	91
Improved training and knowledge of clinicians on CPTSD and attachment disorders.	91
Further clarity regarding the differentiation between an event being classified as a CPTSD traumatic event or as an attachment disorder‐type neglect (e.g., emotional abuse versus emotional deprivation).	80

Statements relating to adults	Participants agreement (%)
**Overlapping features in autism and CPTSD**	
Experiences of alexithymia and/or feeling “numb”.	88
Co‐occurring anxiety (e.g., avoidance of certain social situations, feeling unsafe, hypervigilance).	100
Co‐occurring low mood (e.g., rumination, negative thoughts and feelings such as hopelessness, helplessness, shame, worthlessness).	82
Difficulties with sense of identity and self‐esteem. *This can be moderated by age to a degree, and autistic individuals can have a strong sense of identity*. *(P)*	88
Difficulties identifying/labelling own emotions and understanding own emotions. *In CPTSD, this can stem from an inability to develop an awareness or understanding of their own minds and the minds of others and over time this can become more fixed*. *(P)*	95
Difficulties regulating emotions fluctuating between volatile emotions/strong emotional reactions/overwhelming negative emotions and shutdowns. *Emotions may feel intense and uncontrollable in CPTSD*. *(WG2)*	94
Cognitive rigidity (e.g., inflexibility, thinking patterns) and behavioural rigidity (e.g., preference for routine and control). *Rigidity can present as pervasive and global negative self‐concept in CPTSD*. *(WG2)*	81
Difficulties with concentration and executive function.	82
Sleeping disturbances.	82
Sensitivity to specific sensory stimuli and environments.	82
Reduced interest in social interactions. *Autistic individuals may have a desire for social interaction but find it challenging due to difficulties in social communication*. *(WG1)*	81
Difficulties forming and maintaining relationships.	94
Difficulties understanding and interpreting others' emotions and intentions. *This can present as relationship avoidance and/or feeling cut off from others in CPTSD*. *(WG2)*	82
**Differentiating features between autism and CPTSD**	
Autism is lifelong, whereas CPTSD is typically not. *Learned behaviours resulting from early repetitive interpersonal trauma can be difficult to change*. *(WG1)*	100
Autism is characterized as lifelong, whereas if trauma onset occurs later in adulthood, pre‐existing skills developed by individuals can mitigate the development of CPTSD.	81
Difficulties are evident from birth or an early age in autism, whereas they occur following traumatic events in CPTSD. *Autism can first show signs from the ages of 6 to 12 months*. *(WG2)*	82
The need for predictability is related to rigidity and repetitive behaviours in autism, whereas it concerns seeking safety in CPTSD.	81
Re‐experiencing symptoms (e.g., flashbacks, intrusive thoughts, nightmares) are not characteristic of autism, whereas they are of CPTSD.	88
Repetitive and stereotyped behaviours or movements (e.g., stimming) are characteristic of autism, whereas they are not characteristic of CPTSD. *It can be very difficult to differentiate stimming from self‐soothing. (P) Repetitive behaviours in autism tend to be stereotyped, whereas there can be more maladaptive behaviours, such as addictive behaviour in CPTSD*. *(P)*	82
Special interests that are unusual in nature and intensity are characteristic of autism, whereas they are not characteristic of CPTSD.	94
Sensory difficulties can be related to any aspect of daily life in autism, whereas these are initially a reaction to traumatic events, before generalizing to aspects of daily life (if untreated) in CPTSD. *Any sensory stimulation can result in distress due to hyperarousal in CPTSD, as well as more trauma‐specific triggers*. *(P)*	95
Social difficulties are due to neurodevelopmental differences or impairments in autism, whereas they are due to trust difficulties or a lack of interpersonal safety in CPTSD. *Relationship difficulties can also be due to trauma reminders in CPTSD. (WG2) Unpredictability in relationships, past negative interactions, and fear can all impact relationships in CPTSD. (P) Loss of safety in relationships can also be related to issues such as survivor's guilt and distorted cognitions that change a person's view of people and the world in general in CPTSD*. *(P)*	82
**Overlapping features in autism and EUPD**	
Co‐occurring anxiety associated with avoidance.	92
Difficulties identifying own emotions. *Limited emotional awareness in EUPD may come from a lack of validation, not just through mislabelling. Many people with EUPD are detached from their inner emotions*. *(P)*	83
Difficulties regulating emotions fluctuating between passive and intense outbursts.	83
Difficulties with sense of identity (e.g., establishing own identity, stable sense of self).	88
Difficulties with mentalization. *Mentalization deficits are global and consistent in autism. In EUPD, they are evident when emotionally dysregulated, with evidence of skills when calm that can be built upon in therapy. Misguided assumption that someone showing poor mentalization is autistic, even though this occurs in stressful and conflictual contexts*. *(P)*	83
Cognitive rigidity (e.g., black‐and‐white thinking, catastrophic thinking) and behavioural rigidity (e.g., preference for routine, predictability and control).	100
Sensitivity to specific sensory stimuli and environments.	81
Feeling misunderstood by others and/or a sense of not fitting in.	92
Difficulties in forming and maintaining relationships.	92
Difficulties understanding and interpreting others' emotions and intentions. *The understanding can fluctuate depending on interpersonal stressors and life events in EUPD*. *(P)*	92
**Differentiating features between autism and EUPD**	
Difficulties are evident from birth or an early age in autism, whereas they emerge more clearly in adolescence or young adulthood in EUPD.	92
A desire for routine is characteristic of autism, whereas impulsive actions can be characteristic of EUPD.	92
Repetitive and stereotyped behaviours or movements are characteristic of autism (e.g., stimming), whereas they are not characteristic of EUPD.	100
Special interests that are unusual in nature and intensity are indicative of autism, whereas they are not of EUPD.	92
Language difficulties are characteristic of autism, whereas they are not characteristic of EUPD. *Atypical language and/or language difficulties are common in autism but typically occur in the context of co‐occurring speech delay, intellectual disability, or learning disability*. *(WG2)*	83
Hypervigilance to subtle signs signalling disapproval is not characteristic of autism, whereas it is common in EUPD.	83
Difficulties in interactions are due to a limited inherent understanding of social nuances in autism, whereas they are due to the idealization or devaluation of self and others in EUPD.	92
**Overlapping features in CPTSD and EUPD**	
History of adverse experiences (e.g., traumatic events, invalidation, disrupted attachment).	100
Experiences of feeling “empty” and/or “numb”.	87
Co‐occurring anxiety (e.g., hypervigilance to threat, avoidance).	100
Co‐occurring low mood.	93
Negative feelings (e.g., anger, guilt, shame).	100
Difficulties regulating emotions (e.g., volatile and labile emotions, reactivity).	93
Negative cognitions (e.g., about self and others).	100
Difficulties with own sense of self and self‐image.	93
Difficulties coping with uncertainty.	80
Dissociative experiences.	87
Risk of self‐harm, suicide, substance use/addiction and risky behaviours to cope with emotions that feel unmanageable.	93
Impulsive behaviours.	87
Difficulties understanding and interpreting others' emotions and intentions.	80
Difficulties in forming and maintaining relationships.	93
**Differentiating features between CPTSD and EUPD**	
Difficulties occur at any age in CPTSD, whereas they emerge in adolescence or young adulthood in EUPD.	80
Specific fears of coming to harm again are characteristic of CPTSD, whereas there are varied fears (e.g., rejection, abandonment) in EUPD. *CPTSD can include a range of cognitions resulting from the traumatic events shaping beliefs. Some are fear‐based but others may be related to guilt, shame and anger. In EUPD cognitions may be more tied to the sense of self and others*. *(WG2)*	80
Mood is typically stable and negative in CPTSD, whereas it is unstable with rapid mood swings in EUPD.	82
Re‐experiencing symptoms (e.g., flashbacks, intrusive thoughts, nightmares) are characteristic of CPTSD, whereas they are not of EUPD.	93
Relationships are characterized by mistrust in CPTSD, whereas they are characterized by idealization, devaluation and mistrust in EUPD.	82
**Considerations for autism and CPTSD co‐occurrence**	
Autistic adults may perceive and interpret events differently compared to non‐autistic adults (i.e., higher sensitivity and lower threshold for an event to be experienced as traumatic).	100
Due to increased risk, traumatic events history should be assessed routinely during an autism assessment.	93
Autism and CPTSD are not mutually exclusive and can co‐occur.	87
Co‐occurrence of autism and CPTSD is complex to diagnose.	87
**Considerations for autism and EUPD co‐occurrence**	
*	
*There are experiences of professionals not agreeing on if and how autism and EUPD co‐occur. This can result in service users questioning whether their symptoms are due to one condition or two. (WG1) Reliable diagnosis earlier in an individual's life, can reduce the complexity in teasing about autism from EUPD. Often, there are too many opinions and conflicting reports when they can be considered as co‐occurring. Often, we lack developmental history to know about the onset*. *(P)*	
**Considerations for CPTSD and EUPD co‐occurrence**	
EUPD is a risk factor for co‐occurring trauma (e.g., fragile personality traits increase the risk of traumatic events). *“Fragile personality traits” suggests locating the difficulty in the person rather than in their situation. An alternative might be an increased tendency towards impulsive behaviour, unhelpful coping strategies and relationship difficulties may increase the risk of exposure to trauma*. *(WG2)*	93
**Assessment methods for differential diagnosis of autism and CPTSD**	
Comprehensive history taking (clinical interview). *It is essential for history taking to include family history and developmental history*. *(WG2)*	100
Dating the onset of symptoms relative to traumatic events.	100
Referring to diagnostic criteria (e.g., DSM, ICD).	93
Autism measures (e.g., AAA, ADI, ADOS, AQ, CAT‐Q, CATI, DISCO, EQ, MIDGAS, RAADS, Royal College of Psychiatrists Diagnostic Interview Guide for the Assessment of Adults with Autism Spectrum Disorder).	93
Traumatic event exposure and trauma symptoms measures (e.g., ACE Questionnaire, CAPS, IES, ITI, PCL, PSSI SIDES, TRS).	100
CPTSD measures (e.g., ITI, ITQ).	100
Collateral information from informants (e.g., clinical interviews, reports).	100
Observation of adults during the clinical interviews.	100
Discussion with a multi‐disciplinary team.	93
**Assessment methods for differential diagnosis of autism and EUPD**	
Comprehensive history taking (clinical interview). *It is essential for history taking to include family history and developmental history*. *(WG2)*	100
Autism measures (e.g., AAA, ADI, ADOS, AQ, CAT‐Q, CATI, DISCO, EQ, MIDGAS, RAADS, Royal College of Psychiatrists Diagnostic Interview Guide for the Assessment of Adults with Autism Spectrum Disorder).	92
Collateral information from informants (e.g., clinical interviews, reports).	100
Observation of adults during the clinical interview.	92
Discussion with a multi‐disciplinary team.	92
**Assessment methods for differential diagnosis of CPTSD and EUPD**	
Comprehensive history taking (clinical interview). *Attachment history and evidence of disrupted attachment in earlier life, regardless of later adult life traumas*. *(P)*	100
Dating the onset of symptoms relative to traumatic events.	93
Referring to diagnostic criteria (e.g., DSM, ICD).	93
CPTSD measures (e.g., ITI, ITQ).	86
Collateral information from informants (e.g., clinical interviews, reports).	100
Observation of adults during the clinical interview.	100
Discussion with a multi‐disciplinary team.	100
**Difficulties in distinguishing autism and CPTSD**	
Information provided by adults and informants is not always complete (e.g., informants are unreliable, absence of developmental history, no clear onset of difficulties, unable to express difficulties, language barrier).	93
Autistic adults can learn to mask their difficulties, which may complicate the differentiation of autism and CPTSD.	95
Autism is less recognized and underdiagnosed in females.	86
Cultural norms and expectations influence interactions and social communication, the perception of traumatic events and resilience, help‐seeking and expression of distress.	86
Access to assessment is unequal for those who experience marginalization which may cause an escalation in presentation.	95
Collateral information (e.g., school reports) may reflect societal prejudice.	86
Clinicians do not have adequate training, knowledge or confidence on autism in adult mental health settings.	100
Adult autism services have long wait lists, so adults are not seen in a timely way by the appropriate service.	93
**Suggestions for improved distinction between autism and CPTSD**	
Additional time to assess and/or diagnose (e.g., multiple appointments, comprehensive history and formulation).	93
Review as much collateral information as possible (e.g., school reports).	93
Access to specialist supervision or consultation services of autism and/or CPTSD for assessing clinicians.	100
Improved training and knowledge of clinicians on autism and/or CPTSD.	100
Inclusion of items exploring traumatic events in autism‐specific assessments (e.g., DISCO, ADI) or use of trauma measures in all autism assessments.	90
Development of guidelines and/or tools for differential diagnosis.	93
Further research on differential diagnosis.	87
**Difficulties in distinguishing autism and EUPD**	
Information provided by adults and informants is not always complete (e.g., informants are unreliable, absence of developmental history, no clear onset of difficulties, language barrier).	100
Autistic adults can learn to mask their difficulties, which may complicate the differentiation of autism and EUPD.	81
Autism is less recognized and underdiagnosed in females.	83
Cultural norms and expectations influence help‐seeking and expression of distress.	81
Adults and/or clinicians view an autism diagnosis as more favourable (e.g., increased autism awareness, the stigma of EUPD).	92
Clinicians do not have adequate training, knowledge or confidence on autism in adult mental health settings.	92
Clinicians do not recognize the subtle aspects of autism.	83
Clinicians can hold unfounded subjective views about autism and EUPD, that can impact on the assessment, formulation and diagnostic decisions. *Professionals can attribute introversion, detachment, anankastia, and negative affectivity solely to autism*. *(P)*	88
Adult autism services have long wait lists, so adults are not seen by the appropriate services.	92
**Suggestions for improved distinction between autism and EUPD**	
Additional time to assess and/or diagnose (e.g., multiple appointments, comprehensive history and formulation, postponing diagnosis until clearer picture).	100
Improved training and knowledge of clinicians on autism and/or EUPD.	87
Access to specialist supervision or consultation services on autism and/or EUPD for assessing clinicians.	93
Integration of neurodevelopmental services and mental health services.	87
Avoid over‐reliance on one single tool or measure.	87
Development of guidelines and/or tools for differential diagnosis.	100
Further research on presenting characteristics of co‐occurring autism and EUPD.	87
Further research on differential diagnosis.	87
**Difficulties in distinguishing CPTSD and EUPD**	
Adverse life events can be an aetiological factor for both CPTSD and EUPD.	93
Information provided by adults is not always complete (e.g., absence timeline of difficulties, no clear onset of difficulties).	82
CPTSD is less stigmatizing than EUPD (e.g., placing the problem in traumatic events versus placing the problem in the person, treatable, more hopeful trajectory).	80
EUPD is less recognized and underdiagnosed in males.	88
Cultural norms and expectations influence help‐seeking and expression of distress.	87
Clinicians do not have adequate training, knowledge or confidence on CPTSD.	87
Clinicians are unfamiliar with CPTSD as it is a new diagnosis.	87
**Suggestions for improved distinction between CPTSD and EUPD**	
Additional time to assess and/or diagnose (e.g., comprehensive history and formulation).	87
Improved training and knowledge of clinicians on CPTSD and/or EUPD.	93
Compassionate communication about the development of EUPD as a natural response to an invalidating environment to help challenge stigma.	80
Improved access to records between providers and/or improved record keeping.	87
Avoid endorsing poorly formulated reports as evidence for diagnosis.	82
Improved diagnostic criteria (e.g., clarity and reliability).	87
Development of guidelines and/or tools for differential diagnosis.	93
Further research on differential diagnosis.	87

Items that received ≥80% of professionals' agreement in the last round they were issued in. * No statements in this section. Text in italics offers qualitative comments made by participants (P), experts by experience working group (WG1) and clinicians or researchers working group (WG2). See Supplementary S5 for acronym.

### Overlapping and differentiating features

**Autistic young people and young people with CPTSD** were reported to share several presenting difficulties related to emotional, cognitive, behavioural, sensory and interpersonal functioning. Differences between autistic young people and those with CPTSD described by participants included family history, onset and lifespan of the condition. The nature of repetitive behaviours, sensory sensitivity, and interpersonal challenges helped professionals differentiate between the two conditions. Condition‐specific features were also described, including unusual interests in autism and trauma‐related re‐experiencing symptoms in CPTSD.

**Autistic young people and young people with attachment disorders** were described to share similarities including difficulties related to emotional, behavioural, sensory, interpersonal functioning and sense of self. Differentiating aspects described by participants included differing aetiologies and the nature of interpersonal difficulties. Participants suggested differentiation can also be made based on the presence or absence of autism‐specific features including stereotyped behaviours and unusual interests.

**Young people with attachment disorders and young people with CPTSD** were said to share similarities in terms of a history of adverse experiences, emotional and interpersonal functioning. Professionals differentiated the two by the onset and presence of CPTSD‐specific symptoms (i.e., re‐experiencing).

**Autistic adults and adults with CPTSD** were reported to share similar characteristics including difficulties with emotional, cognitive, behavioural, sensory, interpersonal functioning, and sense of self. The onset and lifespan of the condition helped differentiate between autism and CPTSD. Additionally, the nature of the need for predictability, the type of sensory difficulties, interpersonal challenges and features specific to each condition further supported the differential diagnosis (i.e., unusual interests and repetitive behaviours with autism and re‐experiencing with CPTSD).

**Autistic adults and adults with EUPD** were noted to share several similar features, including difficulties related to emotional, cognitive, behavioural, sensory, interpersonal functioning, and sense of identity. Participants distinguished autism from EUPD by the onset, and the nature of interpersonal challenges. The presence or absence of autism‐specific features (e.g., repetitive and stereotyped behaviours, unusual interests, tendency for routine), and EUPD‐specific features (e.g., impulsivity) further helped professionals distinguish autism from EUPD.

**Adults with EUPD and adults with CPTSD** were said to share many similarities, including a history of adverse experiences, difficulties related to sense of identity, and emotional, behavioural and interpersonal functioning. The onset, nature of fears, mood, and interpersonal difficulties helped professionals differentiate between the two conditions. The presence or absence of specific aspects of CPTSD (e.g., re‐experiencing) further helped differentiate it from EUPD.

### Co‐occurrence considerations

**Young people**. Professionals reported that autism increased vulnerability towards developing CPTSD, and the features of autism may overshadow those of CPTSD. They agreed that autism can co‐occur with DSED, although autistic young people can also experience general difficulties with attachment, without meeting the criteria for attachment disorders. Professionals highlighted that it was crucial to establish the trajectory of features over time to determine whether difficulties are outstanding after an extended period in a stable caregiving environment. Trauma and neglect were identified as shared risk factors for CPTSD and attachment disorders, and participants reported that CPTSD and attachment disorders can co‐occur.

**Adults**. Statements related to co‐occurring autism and EUPD did not reach consensus. Diagnosing co‐occurring autism and CPTSD was reported to be difficult. Professionals noted that autistic adults may interpret traumatic events differently than non‐autistic adults. Routine assessment of traumatic events in autistic adults was described as crucial due to the increased risk of CPTSD in this population. EUPD was described as a risk factor for trauma, possibly leading to co‐occurring CPTSD and EUPD.

### Assessment methods for differential diagnosis

**Young people**. Differential diagnosis methods for all condition pairs included history taking with parents and/or caregivers and young people, the use of condition‐specific measures, reviewing collateral information, observing young people in at least two settings, referring to diagnostic criteria, and discussion with the multi‐disciplinary team. To differentiate between autism and CPTSD, professionals used measures completed by school and/or other professionals. For the differential diagnosis of CPTSD, it was important to determine the onset of symptoms relative to traumatic events. For the differential diagnosis of attachment disorders, professionals conducted observations with parents or caregivers and strangers.

**Adults**. Similarly to young people, in adults, differential diagnosis methods included history taking with the individuals, reviewing collateral information, observation of the individuals and discussion with the multi‐disciplinary team. While autism and CPTSD‐specific measures were used for differential diagnosis, professionals did not report using EUPD‐specific measures. Diagnostic criteria were referred to when differentiating CPTSD from autism or EUPD. As with CPTSD differential diagnosis in young people, in adults, this also involved dating the onset of symptoms relative to traumatic events.

### Difficulties encountered during differential diagnosis

**Young people**. Accurately differentiating between autism, CPTSD and attachment disorders was reported to be difficult due to obtaining incomplete information from young people and informants, influences of cultural norms and expectations, and difficulties recognizing and diagnosing these conditions in certain groups (e.g., autism in females, ethnic minorities, children under care, refugees and asylum seekers). Issues related to professionals who assessed and/or diagnosed these conditions were also described (e.g., adequate training or knowledge). Autism and CPTSD differential diagnosis presented additional challenges because traumatic events may not have a clear onset or may have occurred at a young age. With autism and attachment disorders differential diagnosis, professionals reported there were insufficient measures, and that the diagnostic decision may depend on the services accessed. In the case of CPTSD and attachment disorders, findings showed professionals had difficulties as the conditions can share aetiological factors and both conditions can be mislabelled by professionals as developmental trauma, which is not an existing diagnosis.

**Adults**. When differentiating between autism, CPTSD and EUPD in adults, professionals also faced difficulties such as incomplete information, cultural influences on presentation, difficulty recognizing conditions in certain groups (e.g., autism in females, and EUPD in males), and training or knowledge of professionals. Additional challenges described related to the unfamiliarity of CPTSD diagnosis, the stigma of EUPD, and unequal access to assessment. There was an added difficulty of shared aetiology in terms of experiencing adverse life events when differentiating CPTSD and EUPD.

### Suggestions for improvements

**Young people**. Findings showed that the distinction between autism, CPTSD and attachment disorders can be improved with additional time and multi‐disciplinary team access to assess and diagnose, and better training and knowledge of professionals regarding these conditions. Additionally, there was a need for the development of tools, guidelines and further research. For the differential diagnosis of autism and CPTSD, participants suggested accessing specialist consultation or supervision, to use trauma measures in autism assessments and to improve young people's CPTSD diagnostic criteria. To improve the differential diagnosis of autism and attachment disorders, professionals suggested reviewing as much collateral information as possible, using attachment measures in autism assessments, and avoiding relying on a single tool. For CPTSD and attachment disorders, it was recommended to have improved clarity between events classified as “traumatic” according to CPTSD and “neglect” according to attachment disorders. Concerning attachment disorders differential diagnosis, there was a need for improved awareness of the low prevalence of attachment disorders and the development and use of appropriate attachment disorders‐based tools.

**Adults**. Several areas of suggested improvements were identified by professionals for the differential diagnosis of autism, CPTSD, and EUPD, including more time to assess and diagnose, ensuring professionals have better training and knowledge of these conditions, and developing tools, guidelines, and research on differential diagnosis. For autism and CPTSD differential diagnosis, a thorough review of collateral materials, specialist supervision or consultation and trauma measures in autism assessments were recommended. For autism and EUPD differential diagnosis, possible improvements included access to specialist supervision or consultation, integration of neurodevelopmental and mental health services, not relying on one tool or measure, and research on co‐occurring autism and EUPD. For CPTSD and EUPD differential diagnosis, suggested improvements were reframing EUPD to challenge stigma, better access to records and avoiding the use of poorly formulated reports.

### Working groups' reflections

**Experts by experience** reported that the findings seemed logical and reflected the realities of the human condition. They noted that participants had varying agreement levels with statements (e.g., 80% vs. 100%) and fewer statements reached consensus in certain sections. This mirrored their personal experiences in clinical settings with professionals often having predominant expertise in one area. They reported occasions where professionals held stereotypical or rigid views and had difficulties understanding the nuances of their presentations. Experts by experience emphasized that none of these statements should be used to definitively rule in or out a diagnosis and should only be considered as part of a comprehensive assessment. They also highlighted that professionals sometimes assume that all aspects of diagnostic criteria must apply to an individual. However, this is not always the case as experts by experience shared their experiences of learning to adapt behaviours and needs over time to better fit into societal norms. Thus, some features may appear less obvious. In addition, they emphasized that professionals must check their interpretations with the individuals they assess and/or diagnose to gain insight into the internal functions of behaviours. They stressed that professionals should not make inferences based solely on observable behaviours, as there could be many underlying reasons for any given behaviour.

**Clinicians and researchers** commented that the results were interesting and informative. However, they also noted that the participants seemed to have based their answers on general diagnosis principles rather than on individuals they worked with. This led to a lack of nuance and clinical examples. They found differentiating features more helpful than overlapping features. Although some of the overlapping features identified are diagnostic criteria, clinicians and researchers also emphasized that not all of the other features are necessary for diagnosis. Thus, these features alone must not be used to rule in or rule out a diagnosis, otherwise there is a risk of misdiagnosis.

## DISCUSSION

This study sought to explore professionals' perspectives on the differential diagnosis of autism, CPTSD and attachment disorders in young people and of autism, CPTSD and EUPD in adults. Professionals reported that individuals with any of these conditions, across both age groups, commonly present with difficulties related to emotions (insight and regulation), rigidity (cognitive and behavioural), sensory processing, and interpersonal aspects. Thus, these features do not appear to be reliable indicators of any single diagnosis. Understanding the aetiology and onset, the nature of interpersonal difficulties and identifying condition‐specific features (e.g., unusual interests, re‐experiencing) can help professionals accurately differentiate between these conditions. These results mirror those of previous literature suggesting the nature of the social difficulties and characteristics unique to autism (non‐verbal communication or language difficulties, restricted interests and insistence on sameness), can help distinguish autism and attachment disorders (Zeanah et al., 2016) and EUPD (Lai & Baron‐Cohen, 2015). Individual statements are summaries of clinical experience and will not apply to all individuals with a given condition, as such they should be viewed as supplementary considerations to aid in differentiating complex presentations.

Findings showed that a comprehensive assessment requires taking a detailed history from the individual, as well as their parents and/or caregivers for young people. This also involves measures and observations, reviewing collateral information, referring to diagnostic criteria, and a discussion with a multi‐disciplinary team, which are crucial to differentiate between autism and adversity and/or trauma‐related conditions (Ng‐Cordell et al., 2022). Interestingly, while clinical judgement combined with assessment methods can be useful in clinical practice (Stavropoulos et al., 2018), it was not mentioned by participants. Other authors have highlighted that if diagnostic uncertainty remains, it can be useful to list the features indicative of autism, those suggestive of adversity‐related conditions and the nature of each feature should then be explored (Wilkinson et al., 2023).

While this study focused on the differential diagnosis of individual conditions, a high degree of co‐occurrence has been evidenced in the literature. Although the co‐occurrence of autism and CPTSD remains understudied, there are high rates of PTSD in autistic adults (Quinton et al., 2024; Rumball et al., 2024). Similarly, high co‐occurrence has been reported between autism and attachment disorders (Minnis et al., 2020; Talmón‐Knuser et al., 2023), EUPD and CPTSD (Atkinson et al., 2024), and EUPD and autism (Gillett et al., 2023). Although rates of co‐occurrence between CPTSD or PTSD and attachment disorders are not documented, co‐occurrence has been reported (Zeanah et al., 2016). Given the rates of co‐occurrence among these conditions, the differential diagnosis process should include the identification of co‐occurrence. Failure to do so can result in diagnostic overshadowing, where less obvious conditions may be inaccurately assessed or overlooked due to more apparent diagnoses, resulting in adequate or delayed support and unmet needs (Duvall et al., 2022). As outlined by Karatzias et al. (2023), a hierarchical diagnostic method should be considered, especially in instances of co‐occurring conditions. If a feature is common to two conditions, it can be considered as part of the primary diagnosis, but not used to meet criteria of a co‐occurring condition. Furthermore, as autistic individuals are at greater risk of experiencing traumatic events (Rumball et al., 2021) and there are high rates of neurodevelopmental conditions among young people who have been maltreated (Wilkinson et al., 2023), it is recommended that professionals avoid conducting assessments in silos, based on their respective service or area of expertise. Neurodevelopmental and trauma‐related conditions should both be considered during the assessment of either entity. Although it may be time‐consuming to assess multiple conditions systematically, professionals may consider screening for other conditions and then exploring possible overlapping diagnoses in depth.

Professionals reported differential diagnosis being complicated by incomplete information provided by individuals and informants, regardless of age or condition (e.g., absence of developmental history, no clear onset). Other challenges described by participants were related to social, gender, and ethnicity characteristics of assessed individuals, inequalities in service access and under‐recognition of conditions in certain groups. Large‐scale surveys of professionals have shown that clinicians are less likely to diagnose PTSD in young people in care, in favour of conceptualization as 'developmental trauma' or 'attachment problems' (McGuire et al., 2022). Similar biases exist for gender and ethnicity. For instance, in females, autism is less frequently diagnosed (Loomes et al., 2017) and EUPD is more often diagnosed (Ali & Adshead, 2022). Ethnic minoritized individuals have also been found to be misdiagnosed with mental health difficulties due to differences in presentation or inaccurate diagnoses given (Liang et al., 2016), and autism is under‐identified (Tromans et al., 2021). Overall, a lack of professional training or knowledge about the varying presentations of conditions was reported by participants. Suggestions for improvement included allowing more time for assessment and diagnosis, involving a multi‐disciplinary team, and improving professionals' training and knowledge across mental health conditions and neurodiversity.

### Limitations

Participants from various professions and countries were recruited, but the majority were psychologists from Western countries, which limits the applicability of the findings to non‐Western individuals. Psychologists often rely on formulation rather than attributing difficulties to specific diagnoses, which may influence their approach to differential diagnosis and introduce bias in responses. As participants were international, some may have been unfamiliar with ICD terms, potentially impacting their understanding of the conditions. However, definitions were provided at the start of the survey to reduce the impact of such differences. Additionally, there were fewer participants with expertise in attachment disorders and EUPD than planned, so findings on these conditions represent a small sample of professionals' views. While the majority of participants worked with more than one explored condition, not all of them had experience working across the different conditions, and self‐rated knowledge levels were the lowest for attachment disorders and CPTSD in young people. Although participants were given the option to select neutral answers (i.e., “I don't know”), some participants with less relevant experience may have answered survey questions, which could have impacted the validity of the related statements. Qualitative data analysis of Round 1 responses revealed that participants were sometimes confused between attachment disorders as conceptualized by ICD‐11 (World Health Organization, 2019) and attachment styles (i.e., relational patterns based on Bowlby's attachment theory, 1988); and between ICD‐11 CPTSD and Herman's (1992) “complex trauma” describing a complex form of PTSD not included in diagnostic classifications. This could further impact the validity of the statements regarding these domains.

Although an effort was made to recruit professionals with knowledge and experience on the topic, the statements reflect the perspectives of the participating professionals and are highly dependent on their level of training and professional backgrounds. As a result, some ecologically valid and evidence‐based statements may have not reached consensus, while statements that are at odds with current diagnostic conceptualizations of conditions may have been endorsed. For instance, autism is currently an exclusionary criterion for attachment disorders (World Health Organization, 2019), but most professionals did not agree with these statements. Thus, the consensus statements depict how professionals think in practice when dealing with differential diagnosis dilemmas as diagnostic classifications may not always be sufficient. Although participants could indicate their agreement with parts of the statements, they may have simply agreed or disagreed with full statements instead of noting nuanced aspects. For example, although dissociation is not necessary for a diagnosis of CPTSD, it is common for young people with CPTSD to report dissociative experiences (Haselgruber et al., 2021). Yet, the statement related to dissociation in CPTSD, as well as disengagement in bodily signals in autism, was not endorsed.

Finally, while the study did not focus on co‐occurrence, it is important to note that all of the conditions explored can be diagnosed as co‐occurrences. Although ICD‐11 states that autism and attachment disorders cannot co‐occur (World Health Organization, 2019), research has shown that they can (Minnis et al., 2020; Talmón‐Knuser et al., 2023). Additionally, autism as a diagnosis of exclusion for attachment disorders has been criticized as there is no theoretical reason why autism and an adversity‐related condition cannot co‐occur in young people (Minnis et al., 2020). Therefore, it is also crucial to consider potential co‐occurrence(s) during differential diagnosis to develop a holistic and individualized understanding of an individual's presentation and needs, to inform recommendations and treatment planning.

Currently, there is a move towards neuroaffirmative language in autism (Chetan, 2024). The present study focused on difficulties instead of differences or strengths, due to the nature of diagnostic decisions. The experts by experience also highlighted the importance of naming and acknowledging these difficulties to provide recognition and the necessary support, rather than framing them solely as differences.

### Implications and future research

#### Treatment implications

With differentiating features and suggestions for improved differential diagnosis, it is hoped that the accuracy of diagnosis will increase, leading to reduced occurrences of misdiagnosis and better access to appropriate care. Furthermore, the overlapping features shared across conditions identified in this study could be the basis for transdiagnostic therapy models. Several well‐established interventions already exist for trauma and adversity‐related conditions, including trauma‐focused therapies such as Cognitive Behavioural Therapy, Eye Movement Desensitization and Reprocessing, and modular approaches for CPTSD (Karatzias et al., 2023); Dialectical Behaviour Therapy, Schema‐Focused Therapy, Mentalization‐Based Therapy for EUPD (Morgan & Zimmerman, 2014); and enhanced caregiving for attachment disorders (Zeanah & Gleason, 2015). However, psychological interventions aiming to address transdiagnostic processes, as opposed to condition‐specific difficulties, also have emerging evidence (Mansell et al., 2009). Thus, shared features related to emotions, rigidity, sensory processing, and interpersonal aspects could be the basis for the development of a transdiagnostic therapy model. Finally, the high number of overlapping features across conditions raises questions about the usefulness of diagnosis, supporting the criticisms of psychiatric diagnoses and raising concerns about the current diagnosis‐led service structures. It may be beneficial to adopt a transdiagnostic approach focusing on processes or to view the variations in symptomatology in a dimensional manner, rather than attempting to compartmentalize diagnoses (Jablensky, 2016).

#### Professionals

The findings can support professionals to better understand the similarities between conditions. This can improve their awareness of other conditions than those they work with and encourage them to consider alternative diagnoses during assessments. Professionals can also use the findings to make informed decisions about differential diagnosis, challenge their biases, and identify knowledge gaps. Furthermore, many individuals display atypical presentations, resulting in difficulties assessing and diagnosing, even for experienced professionals (Aboraya et al., 2006). As emphasized by the experts by experience, professionals need to consult individuals they assess to ensure that the diagnosis accurately captures their experiences. Professionals may focus on certain features, while overlooking others, due to their background and training, which can influence their interpretation (Aboraya et al., 2006). Additionally, high workload and time constraints can further affect the accuracy of diagnostic decisions. To address this, the present consensus was that professionals may require further training, teaching and supervision to develop knowledge and clinical curiosity outside of their area of expertise.

#### Services

Most mental health services and training programmes are siloed according to diagnoses, which results in professionals working in one area. Professionals can be encouraged to learn from colleagues in other services and specializations. For example, in trauma services, it is not standard to ask about family or developmental history as it is in autism services. It could be useful to do so to distinguish between conditions or identify underlying neurodevelopmental conditions, which may inform a more tailored treatment approach. Although joint working in mental health services can be complicated, integrated provision can be effective. For instance, Freeman and Peck (2006) found that a specialist partnership of mental health and learning disability services provided opportunities for learning across professions and the exploration of multiple professional perspectives. Rather than having separate services for trauma, attachment disorders, personality disorders and autism, there is a need for integrated services, joint working and/or for professionals with specialist expertise across conditions. This could be achieved through supervision, consultation, split roles across services, joint assessments or discussions on these shared issues.

#### Research

To address the lack of clinical nuances in the statements, it would be beneficial to conduct follow‐up qualitative research such as interviews with professionals, and, importantly, service users, to capture specific examples and complexities of the differential diagnosis of these conditions. Alternatively, case series could further expand the present findings, especially in the case of co‐occurring conditions and complexity. This study highlighted common difficulties encountered by professionals, including insufficient knowledge regarding the presentation of certain conditions in particular groups such as autism in females, autism in ethnic and cultural minoritized groups, and EUPD in males. Research into how conditions present in specific demographic groups is necessary. Participants reported the complexity of diagnosing co‐occurring autism and CPTSD and the need for research on presenting features of co‐occurring autism and EUPD. Further research on the co‐occurrence of these conditions is required. Additionally, this study did not explore the differential diagnosis of emerging EUPD in adolescence. However, literature has suggested that features can manifest in childhood, although they are not typically diagnosed until adulthood (Bach & Vestergaard, 2023). Thus, further research exploring the differential diagnosis of EUPD traits in young people is warranted. Future research could also validate the current study's statements in quantitative population studies to develop appropriate differential diagnosis tools or validated measures. Finally, trait symptom measures could be used in clinical and general population samples to investigate shared and distinct underlying psychological mechanisms and associations between the four conditions.

## CONCLUSION

To the best of the authors' knowledge, this study is the first to explore the distinction between autism, attachment disorders, CPTSD, and EUPD. Consensus statements demonstrate that experts view individuals diagnosed with these conditions as sharing many similar features, which can make accurate differential diagnosis difficult. The findings also provide consensus on differentiating features and assessment methods that can aid professionals in making an accurate differential diagnosis. While this Delphi study provides valuable insights and implications, it also reveals the need for further research and training in this area, and professional collaboration and knowledge sharing across specialities.

## AUTHOR CONTRIBUTIONS

**Rachel Sarr:** Conceptualization; investigation; writing – original draft; methodology; writing – review and editing; formal analysis; project administration; funding acquisition; data curation. **Debbie Spain:** Conceptualization; investigation; methodology; supervision; formal analysis. **Alice M. G. Quinton:** Methodology; funding acquisition; writing – review and editing; conceptualization. **Francesca Happé:** Conceptualization; methodology; writing – review and editing. **Chris R. Brewin:** Conceptualization; methodology; writing – review and editing. **Jonathan Radcliffe:** Conceptualization; methodology; writing – review and editing. **Sally Jowett:** Conceptualization; methodology; writing – review and editing. **Sarah Miles:** Conceptualization; methodology; writing – review and editing. **Rafael A. González:** Conceptualization; methodology; writing – review and editing. **Idit Albert:** Conceptualization; methodology; writing – review and editing. **Alix Scholwin:** Conceptualization; methodology; writing – review and editing. **Marguerite Stirling:** Conceptualization; methodology; writing – review and editing. **Sarah Markham:** Conceptualization; methodology; writing – review and editing. **Sally Strange:** Conceptualization; methodology. **Freya Rumball:** Conceptualization; investigation; methodology; writing – review and editing; formal analysis; supervision.

## FUNDING INFORMATION

This research was funded by RS' Doctorate in Clinical Psychology research grant from King's College London, and AMGQ's PhD research grant. AMGQ is funded by the Medical Research Council (MRC) and King's College London. FH is funded in part by the National Institute for Health Research (NIHR) Maudsley Biomedical Research Centre and King's College London.

## CONFLICT OF INTEREST STATEMENT

The authors declare that there is no conflict of interest.

## ETHICS STATEMENT

The research obtained ethical clearance from King's College London (MRSP‐22/23–34,011) on 29/09/2022. Participants were provided with information sheets and gave written informed consent online.

## Supporting information


Data S1.

## Data Availability

Participants' answers are accessible through a request to the corresponding author.

## Footnotes

^1^
CPTSD was formally defined by the ICD‐11 Working Group in 2013, included in a diagnostic classification for the first time. This allows for the identification of a distinct group with increased complexity and functional impairment.

^2^
In ICD‐11, personality disorder shifted from discrete categories to a dimensional approach. The diagnosis is now differentiated according to the severity and, optionally, personality traits (Gaebel et al., 2020). Although ICD‐11 refers to the term “borderline pattern”, EUPD, also known as borderline personality disorder, is still widely used in clinical practice and is the nomenclature used throughout this paper.

^3^
This was due to limited funding resources to translate the survey and participant data.

## References

[bjop12731-bib-0001] Aboraya, A. , Rankin, E. , France, C. , El‐Missiry, A. , & John, C. (2006). The reliability of psychiatric diagnosis revisited: The Clinician's guide to improve the reliability of psychiatric diagnosis. Psychiatry (Edgmont (Pa.: Township)), 3(1), 41–50. 21103149 PMC2990547

[bjop12731-bib-0002] Ali, S. , & Adshead, G. (2022). Just like a woman: Gender role stereotypes in forensic psychiatry. Frontiers in Psychiatry, 13, 840837. 10.3389/fpsyt.2022.840837 35444574 PMC9014176

[bjop12731-bib-0004] Atkinson, J. R. , Kristinsdottir, K. H. , Lee, T. , & Freestone, M. C. (2024). Comparing the symptom presentation similarities and differences of complex posttraumatic stress disorder and borderline personality disorder: A systematic review. Personality Disorders, Theory, Research, and Treatment, 15(4), 241–253. 10.1037/per0000664 38753372

[bjop12731-bib-0005] Au‐Yeung, S. K. , Bradley, L. , Robertson, A. E. , Shaw, R. , Baron‐Cohen, S. , & Cassidy, S. (2019). Experience of mental health diagnosis and perceived misdiagnosis in autistic, possibly autistic and non‐autistic adults. Autism, 23(6), 1508–1518. 10.1177/1362361318818167 30547677

[bjop12731-bib-0006] Bach, B. , & Vestergaard, M. (2023). Differential diagnosis of ICD‐11 personality disorder and autism Spectrum disorder in adolescents. Children, 10(6), 992. 10.3390/children10060992 37371224 PMC10297099

[bjop12731-bib-0007] Bowlby, J. (1988). A Secure Base: Parent‐child attachment and healthy human development. Basic Books.

[bjop12731-bib-0008] Bozzatello, P. , Rocca, P. , Baldassarri, L. , Bosia, M. , & Bellino, S. (2021). The role of trauma in early onset borderline personality disorder: A biopsychosocial perspective. Frontiers in Psychiatry, 12, 721361. 10.3389/fpsyt.2021.721361 34630181 PMC8495240

[bjop12731-bib-0009] Carter, N. , Bryant‐Lukosius, D. , DiCenso, A. , Blythe, J. , & Neville, A. J. (2014). The use of triangulation in qualitative research. Oncology Nursing Forum, 41(5), 545–547. 10.1188/14.ONF.545-547 25158659

[bjop12731-bib-0010] Chetan, S. (2024). Reframing language in mental health discourses: Towards a more humane approach. Indian Journal of Medical Ethics, 9(1), 73–74. 10.20529/IJME.2023.070 38375648

[bjop12731-bib-0012] Coughlan, B. , Van Ijzendoorn, M. H. , Woolgar, M. , Weisblatt, E. J. L. , & Duschinsky, R. (2022). Differentiating “attachment difficulties” from autism Spectrum disorders and attention deficit hyperactivity disorder: Qualitative interviews with experienced health care professionals. Frontiers in Psychology, 12, 780128. 10.3389/fpsyg.2021.780128 35197884 PMC8860234

[bjop12731-bib-0013] Duvall, S. , Armstrong, K. , Shahabuddin, A. , Grantz, C. , Fein, D. , & Lord, C. (2022). A road map for identifying autism spectrum disorder: Recognizing and evaluating characteristics that should raise red or “pink” flags to guide accurate differential diagnosis. The Clinical Neuropsychologist, 36(5), 1172–1207. 10.1080/13854046.2021.1921276 34121610

[bjop12731-bib-0014] Elliott, R. , McKinnon, A. , Dixon, C. , Boyle, A. , Murphy, F. , Dahm, T. , Travers‐Hill, E. , Mul, C. , Archibald, S. , Smith, P. , Dalgleish, T. , Meiser‐Stedman, R. , & Hitchcock, C. (2021). Prevalence and predictive value of ICD‐11 post‐traumatic stress disorder and complex PTSD diagnoses in children and adolescents exposed to a single‐event trauma. Journal of Child Psychology and Psychiatry, 62(3), 270–276. 10.1111/jcpp.13240 32343370 PMC7984249

[bjop12731-bib-0016] Flackhill, C. , James, S. , Soppitt, R. , & Milton, K. (2017). The Coventry grid interview (CGI): Exploring autism and attachment difficulties. Good Autism Practice, 18(1), 62–80.

[bjop12731-bib-0017] Freeman, T. , & Peck, E. (2006). Evaluating partnerships: A case study of integrated specialist mental health services. Health & Social Care in the Community, 14(5), 408–417. 10.1111/j.1365-2524.2006.00658.x 16918833

[bjop12731-bib-0018] Fusar‐Poli, L. , Brondino, N. , Politi, P. , & Aguglia, E. (2022). Missed diagnoses and misdiagnoses of adults with autism spectrum disorder. European Archives of Psychiatry and Clinical Neuroscience, 272(2), 187–198. 10.1007/s00406-020-01189-w 32892291 PMC8866369

[bjop12731-bib-0019] Gaebel, W. , Stricker, J. , & Kerst, A. (2020). Changes from ICD‐10 to ICD‐11 and future directions in psychiatric classification. Dialogues in Clinical Neuroscience, 22(1), 7–15. 10.31887/DCNS.2020.22.1/wgaebel 32699501 PMC7365296

[bjop12731-bib-0020] Gillett, G. , Leeves, L. , Patel, A. , Prisecaru, A. , Spain, D. , & Happé, F. (2023). The prevalence of autism spectrum disorder traits and diagnosis in adults and young people with personality disorders: A systematic review. The Australian and New Zealand Journal of Psychiatry, 57(2), 181–196. 10.1177/00048674221114603 35986511 PMC9896258

[bjop12731-bib-0021] Gordon, C. , & Lewis, M. (2020). Differentiating between borderline personality disorder and autism spectrum disorder. Mental Health Practice, 23(3), 22–26. 10.7748/mhp.2020.e1456

[bjop12731-bib-0022] Haselgruber, A. , Knefel, M. , Sölva, K. , & Lueger‐Schuster, B. (2021). Foster children's complex psychopathology in the context of cumulative childhood trauma: The interplay of ICD‐11 complex PTSD, dissociation, depression, and emotion regulation. Journal of Affective Disorders, 282, 372–380. 10.1016/j.jad.2020.12.116 33421865

[bjop12731-bib-0023] Hasson, F. , Keeney, S. , & McKenna, H. (2000). Research guidelines for the Delphi survey technique. Journal of Advanced Nursing, 32(4), 1008–1015. 10.1046/j.1365-2648.2000.t01-1-01567.x 11095242

[bjop12731-bib-0024] Herman, J. L. (1992). Trauma and recovery. Basic Books/Hachette Book Group.

[bjop12731-bib-0025] Hsu, C.‐C. , & Sandford, B. A. (2007). The Delphi technique: making sense of consensus. Practical Assessment, Research and Evaluation, 12(10), 1‐8. 10.7275/PDZ9-TH90

[bjop12731-bib-0026] Hyland, P. , Karatzias, T. , Shevlin, M. , McElroy, E. , Ben‐Ezra, M. , Cloitre, M. , & Brewin, C. R. (2021). Does requiring trauma exposure affect rates of ICD‐11 PTSD and complex PTSD? Implications for DSM‐5. Psychological Trauma Theory Research Practice and Policy, 13(2), 133–141. 10.1037/tra0000908 32915045

[bjop12731-bib-0027] Jablensky, A. (2016). Psychiatric classifications: Validity and utility. World Psychiatry, 15(1), 26–31. 10.1002/wps.20284 26833601 PMC4780305

[bjop12731-bib-0028] Jünger, S. , Payne, S. A. , Brine, J. , Radbruch, L. , & Brearley, S. G. (2017). Guidance on conducting and REporting DElphi studies (CREDES) in palliative care: Recommendations based on a methodological systematic review. Palliative Medicine, 31(8), 684–706. 10.1177/0269216317690685 28190381

[bjop12731-bib-0029] Karatzias, T. , Bohus, M. , Shevlin, M. , Hyland, P. , Bisson, J. , Roberts, N. , & Cloitre, M. (2023). Distinguishing between ICD‐11 complex post‐traumatic stress disorder and borderline personality disorder: Clinical guide and recommendations for future research. British Journal of Psychiatry, 223(3), 403–406. 10.1192/bjp.2023.80 37381070

[bjop12731-bib-0030] Kentrou, V. , Oostervink, M. , Scheeren, A. M. , & Begeer, S. (2021). Stability of co‐occurring psychiatric diagnoses in autistic men and women. Research in Autism Spectrum Disorders, 82, 101736. 10.1016/j.rasd.2021.101736

[bjop12731-bib-0031] Lai, M.‐C. , & Baron‐Cohen, S. (2015). Identifying the lost generation of adults with autism spectrum conditions. The Lancet Psychiatry, 2(11), 1013–1027. 10.1016/S2215-0366(15)00277-1 26544750

[bjop12731-bib-0032] Liang, J. , Matheson, B. E. , & Douglas, J. M. (2016). Mental health diagnostic considerations in racial/ethnic minority youth. Journal of Child and Family Studies, 25(6), 1926–1940. 10.1007/s10826-015-0351-z 27346929 PMC4916917

[bjop12731-bib-0033] Loomes, R. , Hull, L. , & Mandy, W. P. L. (2017). What is the male‐to‐female ratio in autism Spectrum disorder? A systematic review and meta‐analysis. Journal of the American Academy of Child & Adolescent Psychiatry, 56(6), 466–474. 10.1016/j.jaac.2017.03.013 28545751

[bjop12731-bib-0034] Mansell, W. , Harvey, A. , Watkins, E. , & Shafran, R. (2009). Conceptual foundations of the Transdiagnostic approach to CBT. Journal of Cognitive Psychotherapy, 23(1), 6–19. 10.1891/0889-8391.23.1.6

[bjop12731-bib-0035] McGuire, R. , Halligan, S. L. , Meiser‐Stedman, R. , Durbin, L. , & Hiller, R. M. (2022). Differences in the diagnosis and treatment decisions for children in care compared to their peers: An experimental study on post‐traumatic stress disorder. British Journal of Clinical Psychology, 61(4), 1075–1088. 10.1111/bjc.12379 35702815 PMC9796033

[bjop12731-bib-0036] McKenzie, R. , & Dallos, R. (2017). Autism and attachment difficulties: Overlap of symptoms, implications and innovative solutions. Clinical Child Psychology and Psychiatry, 22(4), 632–648. 10.1177/1359104517707323 28530116

[bjop12731-bib-0037] Minnis, H. , Messow, C.‐M. , McConnachie, A. , Bradshaw, P. , Briggs, A. , Wilson, P. , & Gillberg, C. (2020). Autism and attachment disorder symptoms in the general population: Prevalence, overlap, and burden. Developmental Child Welfare, 2(1), 37–51. 10.1177/2516103220902778 PMC13021084 41907538

[bjop12731-bib-0038] Moran, H. (2010). Clinical observations of the differences between children on the autism spectrum and those with attachment problems: The Coventry grid. Good Autism Practice, 11(2), 44–57.

[bjop12731-bib-0039] Morgan, T. A. , & Zimmerman, M. (2014). Is borderline personality disorder underdiagnosed and bipolar disorder Overdiagnosed? In Borderline personality and mood disorders: Comorbidity and controversy (2015th ed., pp. 65–78). Springer.

[bjop12731-bib-0040] Mouchabac, S. , Conejero, I. , Lakhlifi, C. , Msellek, I. , Malandain, L. , Adrien, V. , Ferreri, F. , Millet, B. , Bonnot, O. , Bourla, A. , & Maatoug, R. (2021). Improving clinical decision‐making in psychiatry: Implementation of digital phenotyping could mitigate the influence of patient's and practitioner's individual cognitive biases. Dialogues in Clinical Neuroscience, 23(1), 52–61. 10.1080/19585969.2022.2042165 35860175 PMC9286737

[bjop12731-bib-0041] Ng‐Cordell, E. , Rai, A. , Peracha, H. , Garfield, T. , Lankenau, S. E. , Robins, D. L. , Berkowitz, S. J. , Newschaffer, C. , & Kerns, C. M. (2022). A qualitative study of self and caregiver perspectives on how autistic individuals cope with trauma. Frontiers in Psychiatry, 13, 825008. 10.3389/fpsyt.2022.825008 35911211 PMC9329569

[bjop12731-bib-0042] O'Connor, C. , & McNicholas, F. (2020). Lived experiences of diagnostic shifts in child and adolescent mental health contexts: A qualitative interview study with young people and parents. Journal of Abnormal Child Psychology, 48(8), 979–993. 10.1007/s10802-020-00657-0 32447487

[bjop12731-bib-0043] Porr, V. (2017). Real life consequences of stigmatization, misdiagnosis, misunderstanding, and mistreatment of borderline personality disorder. European Psychiatry, 41(S1), S259–S260. 10.1016/j.eurpsy.2017.02.065

[bjop12731-bib-0044] Quinton, A. M. G. , Ali, D. , Danese, A. , Happé, F. , & Rumball, F. (2024). The assessment and treatment of post‐traumatic stress disorder in autistic people: A systematic review. Review Journal of Autism and Developmental Disorders. 10.1007/s40489-024-00430-9 PMC13002758 41868251

[bjop12731-bib-0045] Rumball, F. , Brook, L. , Happé, F. , & Karl, A. (2021). Heightened risk of posttraumatic stress disorder in adults with autism spectrum disorder: The role of cumulative trauma and memory deficits. Research in Developmental Disabilities, 110, 103848. 10.1016/j.ridd.2020.103848 33454451

[bjop12731-bib-0046] Rumball, F. , Parker, R. , Madigan, A. E. , Happe, F. , & Spain, D. (2024). Elucidating the presentation and identification of PTSD in autistic adults: A modified Delphi study. Advances in Autism, 10(3), 163–184. 10.1108/AIA-08-2023-0053

[bjop12731-bib-0047] Rutter, M. , Kreppner, J. , & Sonuga‐Barke, E. (2009). Emanuel miller lecture: Attachment insecurity, disinhibited attachment, and attachment disorders: Where do research findings leave the concepts? Journal of Child Psychology and Psychiatry, 50(5), 529–543. 10.1111/j.1469-7610.2009.02042.x 19298474

[bjop12731-bib-0060] Sarr, R. , Quinton, A. , Spain, D. , & Rumball, F. (2024). A Systematic Review of the Assessment of ICD‐11 Complex Post‐Traumatic Stress Disorder (CPTSD) in Young People and Adults. Clinical Psychology & Psychotherapy, 31(3), 1‐37. Portico. 10.1002/cpp.3012 38894553

[bjop12731-bib-0048] Stavropoulos, K. , Bolourian, Y. , & Blacher, J. (2018). Differential diagnosis of autism Spectrum disorder and post traumatic stress disorder: Two clinical cases. Journal of Clinical Medicine, 7(4), 71. 10.3390/jcm7040071 29642485 PMC5920445

[bjop12731-bib-0049] Stemler, S. (2000). An overview of content analysis. Practical Assessment, Research and Evaluation, 7(17), 1‐6. 10.7275/Z6FM-2E34

[bjop12731-bib-0050] Talmón‐Knuser, F. , González‐Sala, F. , Lacomba‐Trejo, L. , & Samper‐García, P. (2023). Reactive attachment disorder and its relationship to psychopathology: A systematic review. Children, 10(12), 1892. 10.3390/children10121892 38136094 PMC10741566

[bjop12731-bib-0051] Tromans, S. , Chester, V. , Gemegah, E. , Roberts, K. , Morgan, Z. , Yao, G. L. , & Brugha, T. (2021). Autism identification across ethnic groups: A narrative review. Advances in Autism, 7(3), 241–255. 10.1108/AIA-03-2020-0017

[bjop12731-bib-0052] Webster, C. S. , Taylor, S. , & Weller, J. M. (2021). Cognitive biases in diagnosis and decision making during anaesthesia and intensive care. BJA Education, 21(11), 420–425. 10.1016/j.bjae.2021.07.004 34707887 PMC8520040

[bjop12731-bib-0053] Wilkinson, S. , Evans, S. , & DeJong, M. (2023). Assessing autism spectrum disorder in children with a background of maltreatment: Challenges and guidance. Archives of Disease in Childhood, 108(8), 597–600. 10.1136/archdischild-2022-323986 36385007

[bjop12731-bib-0054] Woolgar, M. , & Scott, S. (2014). The negative consequences of over‐diagnosing attachment disorders in adopted children: The importance of comprehensive formulations. Clinical Child Psychology and Psychiatry, 19(3), 355–366. 10.1177/1359104513478545 23575458

[bjop12731-bib-0055] World Health Organization . (2016). International statistical classification of diseases and related health problems (10th ed.). WHO. https://icd.who.int/browse10/2016/en 10.2471/BLT.25.294650 PMC13531092 42683092

[bjop12731-bib-0056] World Health Organization . (2019). International statistical classification of diseases and related health problems (11th ed.). WHO. https://icd.who.int/ 10.2471/BLT.25.294650 PMC13531092 42683092

[bjop12731-bib-0057] World Health Organization . (2024). Clinical descriptions and diagnostic requirements for ICD‐11 mental, behavioural and neurodevelopmental disorders. WHO. https://iris.who.int/bitstream/handle/10665/375767/9789240077263‐eng.pdf?sequence=1

[bjop12731-bib-0059] Zeanah, C. H. , & Gleason, M. M. (2015). Annual research review: Attachment disorders in early childhood – Clinical presentation, causes, correlates, and treatment. Journal of Child Psychology and Psychiatry, 56(3), 207–222. 10.1111/jcpp.12347 25359236 PMC4342270

[bjop12731-bib-0058] Zeanah, C. H. , Chesher, T. , Boris, N. W. , Walter, H. J. , Bukstein, O. G. , Bellonci, C. , Benson, R. S. , Bussing, R. , Chrisman, A. , Hamilton, J. , Hayek, M. , Keable, H. , Rockhill, C. , Siegel, M. , & Stock, S. (2016). Practice parameter for the assessment and treatment of children and adolescents with reactive attachment disorder and disinhibited social engagement disorder. Journal of the American Academy of Child & Adolescent Psychiatry, 55(11), 990–1003. 10.1016/j.jaac.2016.08.004 27806867

