# Supplementary material for: Differential diagnosis of autism, attachment disorders, complex post‐traumatic stress disorder and emotionally unstable personality disorder: A Delphi study
Source: Br J Psychol. 2024 Sep 20;116(1):1–33. doi: 10.1111/bjop.12731 (PMC11724683; doi:10.1111/bjop.12731)
Supplement: Supplementary file 1 — Data S1. [file BJOP-116-1-s001.docx]

Supplementary Materials

1. Additional Demographics

**Table 1**. Additional participants details.

|  | **Round 1**  **(*n)*** | **Round 2**  **(*n)*** | **Round 3**  **(*n)*** |
| --- | --- | --- | --- |
| **Country of clinical training*** |  |  |  |
| Australia | 7 |  |  |
| Canada | 6 |  |  |
| Georgia | 1 |  |  |
| Germany | 1 |  |  |
| Ireland | 5 |  |  |
| Italy | 1 |  |  |
| South Africa | 3 |  |  |
| Spain | 2 |  |  |
| Switzerland | 1 |  |  |
| United Kingdom | 66 |  |  |
| United States of America | 13 |  |  |
| **Age groups worked with*** |  |  |  |
| Children (under 7 years old) | 41 | 28 | 21 |
| Children (7 to 12 years old) | 53 | 34 | 27 |
| Adolescents (13 to 17 years old) | 67 | 41 | 39 |
| Adults (18 years old or older) | 75 | 38 | 36 |
|  | **Round 1  ratings** | **Round 2  ratings** | **Round 3  ratings** |
| **Knowledge and understanding of mental health co-occurring conditions for expertise condition*** |  |  |  |
| Attachment disorders | 9.00 | 8.50 | 8.75 |
| Autism (young people) | 8.49 | 8.40 | 8.50 |
| Autism (adults) | 8.62 | 8.90 | 8.92 |
| CPTSD (young people) | 7.50 | 7.50 | 7.20 |
| CPTSD (adults) | 8.75 | 9.00 | 8.50 |
| EUPD | 9.18 | 9.33 | 9.33 |
| **Knowledge and understanding of differential diagnosis for expertise condition*** |  |  |  |
| Attachment disorders | 8.71 | 8.50 | 8.75 |
| Autism (young people) | 8.57 | 8.48 | 8.50 |
| Autism (adults) | 8.24 | 8.60 | 8.58 |
| CPTSD (young people) | 7.00 | 7.00 | 6.60 |
| CPTSD (adults) | 8.29 | 8.88 | 8.20 |
| EUPD | 8.45 | 9.00 | 9.00 |

* Questions asked at Round 1 only. Self-rated knowledge and understanding are means of ratings from 1 (know very little) to 10 (very well informed).

1. Participation

**Table 2**. Number of participants per category

| Count of Core clinical profession | **Round 2 (*n)*** | **Round 3**  **(*n)*** |
| --- | --- | --- |
| **Statements relating to young people** |  |  |
| Overlapping features in autism and CPTSD | 34 | 24 |
| Differentiating features between autism and CPTSD | 34 | 24 |
| Overlapping features in autism and attachment disorders | 25 | 22 |
| Differentiating features between autism and attachment disorders | 25 | 22 |
| Overlapping features in CPTSD and attachment disorders | 11 | 10 |
| Differentiating features between CPTSD and attachment disorders | 11 | 10 |
| Considerations for autism and CPTSD co-occurrence | 33 | 23 |
| Considerations for autism and attachment disorders co-occurrence | 26 | 21 |
| Considerations for CPTSD and attachment disorders co-occurrence | 11 | * |
| Assessment methods for differential diagnosis of autism and CPTSD | 33 | 23 |
| Assessment methods for differential diagnosis of autism and attachment disorders | 25 | 21 |
| Assessment methods for differential diagnosis of CPTSD and attachment disorders | 11 | 10 |
| Difficulties in distinguishing autism and CPTSD | 32 | 23 |
| Suggestions for improved distinction between autism and CPTSD | 32 | 23 |
| Difficulties in distinguishing autism and attachment disorders | 24 | 21 |
| Suggestions for improved distinction between autism and attachment disorders | 24 | 21 |
| Difficulties in distinguishing CPTSD and attachment disorders | 11 | 10 |
| Suggestions for improved distinction between CPTSD and attachment disorders | 11 | 10 |
| **Statements relating to adults** |  |  |
| Overlapping features in autism and CPTSD | 17 | 21 |
| Differentiating features between autism and CPTSD | 17 | 21 |
| Overlapping features in autism and EUPD | 12 | 16 |
| Differentiating features between autism and EUPD | 12 | 15 |
| Overlapping features in CPTSD and EUPD | 15 | 17 |
| Differentiating features between CPTSD and EUPD | 15 | 17 |
| Considerations for autism and CPTSD co-occurrence | 15 | 21 |
| Considerations for autism and EUPD co-occurrence | 12 | 16 |
| Considerations for CPTSD and EUPD co-occurrence | 15 | * |
| Assessment methods for differential diagnosis of autism and CPTSD | 14 | 21 |
| Assessment methods for differential diagnosis of autism and EUPD | 12 | 16 |
| Assessment methods for differential diagnosis of CPTSD and EUPD | 14 | 17 |
| Difficulties in distinguishing autism and CPTSD | 15 | 21 |
| Suggestions for improved distinction between autism and CPTSD | 15 | 21 |
| Difficulties in distinguishing autism and EUPD | 12 | 16 |
| Suggestions for improved distinction between autism and EUPD | 12 | 15 |
| Difficulties in distinguishing CPTSD and EUPD | 15 | 17 |
| Suggestions for improved distinction between CPTSD and EUPD | 15 | 17 |

* No statements in this section.

1. Questionnaires

**Figure 1.** Items included, excluded and re-rated during Round 1, 2 and 3

* Not including demographics and expertise questions.

1. Non-Consensus Statements

**Table 3**. Statements that did not reach consensus.

| **Statements relating to young people** | **Participants agreement (%)** |
| --- | --- |
| **Overlapping features in autism and CPTSD** |  |
| Co-occurring low mood. | 63 |
| Negative cognitions (e.g., about self, others and the world). | 44 |
| Difficulties with sense of identity. | 59 |
| Risk of self-harm and suicidal behaviour. | 53 |
| Unusual play and interests (e.g., repetitive play, restricted activities). | 38 |
| Repetitive play. | 63 |
| Difficulties with non-verbal communication (e.g., eye contact, facial expressions). | 53 |
| Difficulties with verbal communication (e.g., expressivity, pragmatic). | 53 |
| Reduced motivation, interest and enjoyment in social interactions. | 35 |
| **Differentiating features between autism and CPTSD** |  |
| Autism is lifelong, whereas CPTSD is typically not. | 50 |
| Skills are generally inconsistent with expected traditional milestones in autism, whereas traditional milestones are met in CPTSD. | 75 |
| Stressors can be related to any aspects of daily life in autism, whereas they are related to traumatic events in CPTSD. | 59 |
| Emotional literacy and regulation can be limited in autism, whereas they are not in CPTSD. | 26 |
| Negative cognitions and emotions are not characteristic of autism, whereas they are characteristic of, and related to, traumatic events in CPTSD. | 44 |
| Hyperfocus and disengagement in bodily signals (e.g., hunger, pain) are characteristic of autism, whereas dissociative experiences are characteristic of CPTSD. | 53 |
| Imaginative and varied play can be limited in autism, whereas it is not in CPTSD. | 63 |
| Play with others can be limited in autism, whereas it is not in CPTSD (e.g., able to join others' play, let others join their play). | 26 |
| Triggers are specific to sensory sensitivities in autism, whereas they are specific to reminders of traumatic events in CPTSD. | 75 |
| Interoception difficulties are characteristic of autism, whereas they are not characteristic of CPTSD. | 53 |
| Inherent understanding of others and relationships can be limited in autism, whereas it is not in CPTSD. | 47 |
| **Overlapping features in autism and attachment disorders** |  |
| Co-occurring neurodevelopmental or physical health conditions. | 64 |
| Difficulties with cognitive functioning (e.g., concentration, memory, executive function). | 77 |
| Repetitive behaviours (e.g., self-stimulating). | 56 |
| Impulsive behaviours. | 73 |
| Risk of self-harm. | 50 |
| Unusual play (e.g., differences in the quality of imaginative play, difficulties with collaborative play). | 77 |
| Unusual interests (e.g., restricted). | 40 |
| Unusual attachment to certain possessions or items. | 77 |
| Heightened awareness of the environment (e.g., for threat perception). | 73 |
| Difficulties with verbal communication (e.g., reciprocity). | 77 |
| Limited awareness of strangers and familiarity. | 73 |
| Reduced interest in social interactions. | 52 |
| Difficulties regulating social response that fluctuates between extreme social disinhibition (e.g., inappropriate social boundaries) and inhibition (e.g., social withdrawal). | 77 |
| Social disinhibition towards strangers. | 59 |
| **Differentiating features between autism and attachment disorders** |  |
| Autism is characterised as lifelong, whereas reactive attachment disorder recovers with changes in environmental provision and care. | 73 |
| Autism is lifelong, whereas attachment disorders are typically not. | 24 |
| Co-occurring neurodevelopmental or physical health conditions are characteristic of autism, whereas they are not in attachment disorders. | 20 |
| Emotion regulation difficulties are not related to threats to the relationship or to gaining reactions from caregivers in autism, whereas they are related to feeling safe in attachment disorders. | 56 |
| Eating difficulties can be related to sensory differences or a need for predictability in autism, whereas they are related to hoarding or parental rigidity/control in attachment disorders. | 68 |
| Imaginative and varied play can be limited in autism, whereas it is not in attachment disorders. | 44 |
| Eating difficulties can be related to sensory processing in autism, whereas they are related to hoarding in attachment disorders. | 52 |
| Sensory difficulties can be characteristic of autism, whereas they are not characteristic of attachment disorders. | 32 |
| Sensory-seeking behaviours bring pleasure in autism, whereas they are in response or reaction to others in attachment disorders. | 52 |
| Language difficulties can be characteristic of autism, whereas they are not characteristic of attachment disorders. | 48 |
| Social motivation and interest can be limited in autism, whereas they are not in attachment disorders. | 36 |
| There is no noticing of subtle signs of distress in others with autism, whereas there is hypervigilance to them in attachment disorders. | 44 |
| Excessive reassurance seeking for bonding is not characteristic of autism, whereas it is characteristic of attachment disorders. | 48 |
| The ability to take others' perspectives can be limited in autism, whereas it is not in attachment disorders (e.g., aiming to create a good impression). | 36 |
| Inherent social understanding can be limited in autism, whereas it is not in attachment disorders. | 52 |
| Patterns of interactions are consistent and pervasive in autism, whereas they depend on people and situations in attachment disorders. | 48 |
| Reciprocal interaction can be limited in autism, whereas it is not in attachment disorders. | 32 |
| **Overlapping features in CPTSD and attachment disorders** |  |
| Co-occurring anxiety (e.g., fear, hypervigilance, avoidance). | 50 |
| Co-occurring low mood associated with negative cognitions. | 60 |
| Difficulties understanding own emotions. | 60 |
| Difficulties with concentration. | 60 |
| Rigid behaviours (e.g., preference for predictability and control). | 54 |
| Reduced interest and enjoyment in social interactions. | 36 |
| Difficulties understanding others' emotions and intentions. | 70 |
| Difficulties regulating social relationships, fluctuating between social disinhibition (e.g., intense attachment to another) and inhibition (e.g., social withdrawal). | 55 |
| **Differentiating features between CPTSD and attachment disorders** |  |
| A history of traumatic events is necessary for CPTSD, whereas it is not for attachment disorders. | 55 |
| Exposure to traumatic events can occur at any age in CPTSD, whereas insufficient care must happen by age 5 for reactive attachment disorder, or at any age for disinhibited social engagement disorder. | 30 |
| Difficulties typically persist in CPTSD, whereas a spontaneous recovery can occur when placed in a stable environment in reactive attachment disorder. | 18 |
| The absence of a preferred attachment figure is not necessary for CPTSD, whereas it is in attachment disorders. | 55 |
| Social disinhibition is not characteristic of CPTSD, whereas it is characteristic of attachment disorders. | 18 |
| **Considerations for autism and CPTSD co-occurrence** |  |
| The same behaviour can occur in the context of autism or CPTSD (e.g., “stimming”). | 61 |
| **Considerations for autism and attachment disorders co-occurrence** |  |
| Autism is an exclusionary diagnosis for reactive attachment disorder. | 15 |
| If co-occurrence of autism and attachment disorder is suspected, assessment of autism should be prioritised over assessment of attachment disorder. | 48 |
| **Considerations for CPTSD and attachment disorders co-occurrence** |  |
| * |  |
| **Assessment methods for differential diagnosis of autism and CPTSD** |  |
| Cognitive and/or executive function assessments or measures (e.g., BRIEF, BrownADDScales, DAS, NEPSY, WISC). | 48 |
| Developmental measures (e.g., PEDS). | 65 |
| Sensory profile measures (e.g., Sensory Profile). | 56 |
| Adaptive behaviour measures (e.g., VABS). | 42 |
| Emotional and behavioural functioning measures (e.g., Bar-On EQ, BASC, RSCA, SDQ). | 52 |
| Theory of Mind measures. | 48 |
| **Assessment methods for differential diagnosis of autism and attachment disorders** |  |
| Reactive attachment disorder should be assessed after an autism diagnosis has been considered and ruled out. | 57 |
| Referring to guidance (e.g., AACAP guidance). | 76 |
| Cognitive and/or executive function assessments or measures (e.g., BRIEF, BrownADDScales, DAS, NEPSY, WISC). | 40 |
| Adaptive behaviour measures (e.g., VABS). | 40 |
| Sensory profile measures (e.g., Sensory Profile). | 56 |
| Emotional and behavioural functioning measures (e.g., Bar-On EQ, RSCA). | 56 |
| Projective assessments (e.g., Rorschach). | 20 |
| Differentiating measures (e.g., Coventry Grid). | 76 |
| Attachment patterns differentiating measures (e.g., Coventry Grid). | 76 |
| **Assessment methods for differential diagnosis of CPTSD and attachment disorders** |  |
| Projective assessments (e.g., Rorschach). | 9 |
| **Difficulties in distinguishing autism and CPTSD** |  |
| Autism diagnosis is favoured by families as it is viewed as less stigmatising than CPTSD. | 47 |
| Adolescent females may mistakenly identify as autistic due to masking and variations in the female presentation when they are in a typical developmental phase (e.g., identity exploration, self-doubt), which may complicate the differentiation of autism and CPTSD. | 65 |
| Minoritised and oppressed young people (e.g., gender and sexuality diverse individuals, ethnic minoritised groups) may develop different patterns of thoughts and behaviours to cope in oppressive environments (e.g., hypervigilance) that are misdiagnosed as symptoms of one of the conditions. | 74 |
| Young people of lower socio-economic status are assumed to have CPTSD, and autism is overlooked. | 50 |
| There are insufficient sensitive and specific measures to aid clinical differentiation between autism and CPTSD. | 78 |
| There is limited provision for joint working. | 70 |
| Differential diagnosis decision depends on access to appropriate services following diagnosis. | 70 |
| **Suggestions for improved distinction between autism and CPTSD** |  |
| Improved specificity of autism measures. | 78 |
| **Difficulties in distinguishing autism and attachment disorders** |  |
| Young people’s families and/or clinicians view an autism diagnosis as more favourable (e.g., increased autism awareness, the perceived judgment of attachment disorders). | 52 |
| Autism is favoured by families as it is viewed as less stigmatising than attachment disorders. | 48 |
| Families identify their children as having an autism diagnosis and attachment disorders are overlooked. | 62 |
| Attachment disorders are favoured by families as these are viewed as less stigmatising than autism and autism is overlooked. | 19 |
| Young people of lower socio-economic status are assumed to have attachment disorders and autism is overlooked. | 50 |
| Clinicians do not refer to existing clinical guidance (e.g., AACAP guidance) or diagnostic classifications when considering attachment disorders as a possible diagnosis. | 71 |
| Differential diagnosis decision depends on access to appropriate services following diagnosis. | 67 |
| **Suggestions for improved distinction between autism and attachment disorders** |  |
| Refrain from using tools that assess attachment styles or patterns to assess attachment disorders (e.g., Coventry Grid, CAI). | 38 |
| **Difficulties in distinguishing CPTSD and attachment disorders** |  |
| Attachment disorders may be more noticeable in young people, whereas CPTSD can be more difficult to identify. | 40 |
| Young people who have been abused are assumed to have attachment disorders, and CPTSD is overlooked. | 70 |
| Young people under social care are assumed to have attachment disorders and CPTSD is overlooked. | 55 |
| Available tools for attachment disorders are inappropriate (e.g., too long, do not correlate with attachment disorders but rather attachment styles, involving play leading to misdiagnosis). | 70 |
| **Suggestions for improved distinction between CPTSD and attachment disorders** |  |
| Refrain from using tools that assess attachment styles or patterns to assess attachment disorders (e.g., Coventry Grid, CAI). | 60 |
| Use of DSM-5 criteria for attachment disorders. | 50 |

| **Statements relating to adults** | **Participants agreement (%)** |
| --- | --- |
| **Overlapping features in autism and CPTSD** |  |
| Co-occurring anger. | 53 |
| Feeling disconnected from self and/or dissociative experiences. | 53 |
| Participation in a limited range of activities. | 67 |
| Repetitive behaviours (e.g., self-soothing behaviours). | 76 |
| Impulsive behaviours. | 53 |
| Risk of self-harm, suicidal behaviour and substance use. | 67 |
| Eating disturbances. | 53 |
| Difficulties with non-verbal communication (e.g., eye contact). | 62 |
| Difficulties with verbal communication (e.g., expressivity). | 76 |
| **Differentiating features between autism and CPTSD** |  |
| Emotional literacy can be limited in autism, whereas it is not in CPTSD. | 59 |
| Emotional distress can be inconsistent in autism, whereas it is persistent in CPTSD. | 53 |
| Emotional distress is often specifically linked to sensory overwhelm or change in autism, whereas it is not in CPTSD. | 59 |
| Negative feelings (e.g., lack of enjoyment, fear) are not characteristic of autism, whereas they are characteristic of CPTSD. | 50 |
| Suicidal thoughts are not characteristic of autism, whereas they are characteristic of CPTSD. | 29 |
| Executive function difficulties can be associated with autism, whereas they are not characteristic of CPTSD. | 18 |
| Difficulties with memory and concentration are not characteristic of autism, whereas they are characteristic of CPTSD. | 41 |
| Unconscious periods of hyperfocus and disengagement in bodily signals (e.g., hunger, pain) are characteristic of autism, whereas dissociative experiences are characteristic of CPTSD. | 53 |
| Seeking predictability acts to support executive function difficulties in autism, whereas it is to reduce the fight or flight response in CPTSD. | 71 |
| The need for control is related to rigidity and repetitive behaviours in autism, whereas it is to feel safe in CPTSD. | 47 |
| Hypervigilance is not characteristic of autism, whereas it is characteristic of CPTSD. | 67 |
| Risky behaviours (e.g., substance use, self-harm) are not characteristic of autism, whereas they are characteristic of CPTSD. | 35 |
| Sensory processing difficulties are characteristic of autism, whereas they are not characteristic of CPTSD. | 53 |
| Sensory difficulties can be related to any aspect of daily life in autism, whereas they are related to traumatic events in CPTSD. | 71 |
| Non-verbal communication can be poorly modulated in autism, whereas it is modulated in CPTSD. | 67 |
| Language and social communication difficulties (e.g., idiosyncratic or repetitive language, reciprocity) are characteristic of autism, whereas they are not characteristic of CPTSD. | 76 |
| Relationships feel safe in autism, whereas they can feel unsafe (e.g., fear of abandonment, mistrust, volatile) in CPTSD. | 35 |
| Relationships can feel unsafe due to their unpredictability or past negative interactions in autism, whereas they can feel unsafe due to mistrust or fear in CPTSD. | 71 |
| Inherent understanding of others and relationships can be limited in autism, whereas it is not in CPTSD. | 41 |
| **Overlapping features in autism and EUPD** |  |
| Experiences of alexithymia and/or feeling "numb". | 69 |
| Co-occurring low mood (e.g., negative feelings such as hopelessness, rumination). | 42 |
| Tendency for egocentricity. | 50 |
| Feeling disconnected from self and/or dissociative experiences. | 50 |
| Difficulties with executive function (e.g., planning, organising). | 75 |
| Impulsive behaviours. | 56 |
| Intense passions or interests. | 58 |
| Risk of self-harm, suicidal behaviour and substance use. | 75 |
| Difficulties with non-verbal communication (e.g., eye contact). | 25 |
| Difficulties with verbal communication (e.g., modulating the conversation based on context). | 58 |
| **Differentiating features between autism and EUPD** |  |
| There is a risk of childhood adversity in autism, whereas there are frequent adverse childhood events in EUPD. | 50 |
| Emotional literacy difficulties can be due to difficulties in awareness of the person’s own emotions in autism, whereas they can be due to mislabelling of the person’s own emotions in EUPD. | 53 |
| Emotional literacy can be limited in autism, whereas it is not in EUPD. | 33 |
| Emotion regulation difficulties are due to sensory overwhelm or change in autism, whereas they are due to interpersonal triggers in EUPD. | 58 |
| Mentalisation difficulties can be consistently associated with autism, whereas these occur in the context of emotional dysregulation in EUPD. | 60 |
| Instability in self-esteem and self-image is uncommon in autism, whereas it is common in EUPD. | 33 |
| Seeking predictability acts to support executive function difficulties in autism, whereas it is to reduce the fight or flight response in EUPD. | 67 |
| High-risk behaviours are not characteristic of autism, whereas they are common in EUPD. | 58 |
| Self-harm occurs due to sensory overstimulation in autism, whereas it is due to emotional dysregulation or negative feelings about the self in EUPD. | 53 |
| Self-harm is not an impulsive action in autism, whereas it can be in EUPD. | 33 |
| Sensory processing difficulties are characteristic of autism, whereas they are not characteristic of EUPD. | 42 |
| Non-verbal communication can be poorly modulated in autism, whereas it is not in EUPD. | 58 |
| Non-verbal communication difficulties are characteristic of autism, whereas they are not characteristic of EUPD. | 60 |
| Social motivation and interest can be limited in autism, whereas they are not in EUPD. | 58 |
| Avoidance of social situations is more characteristic of autism, whereas it is not characteristic of EUPD. | 58 |
| Inherent understanding of facial expressions can be limited for all emotions in autism, whereas it is only limited for neutral and negative expressions in EUPD. | 53 |
| Limited inherent understanding of others in autism, whereas there is inconsistent understanding of others in EUPD. | 73 |
| Social interactions lead to burnout in autism, whereas they lead to negative emotions or responses in EUPD. | 58 |
| Relationships are not volatile in autism, whereas they can be in EUPD. | 58 |
| Attempts to elicit care from relationships (e.g., demanding, absence of boundaries) are not common in autism, whereas they are in EUPD. | 58 |
| Fears of abandonment and rejection are not common in autism, whereas they are in EUPD. | 60 |
| Social communication difficulties are characteristic of autism, whereas social difficulties occur due to feeling misunderstood in EUPD. | 67 |
| Limited social reciprocity is characteristic of autism, whereas it is not of EUPD. | 67 |
| **Overlapping features in CPTSD and EUPD** |  |
| Experiencing flashbacks and/or intrusive thoughts/memories. | 65 |
| Fears of abandonment. | 71 |
| **Differentiating features between CPTSD and EUPD** |  |
| Difficulties occur following the exposure to traumatic events in CPTSD, whereas they occur following exposure to invalidating environments in EUPD. | 53 |
| Experiences of betrayal are linked to CPTSD, whereas experiences of abandonment are linked to EUPD. | 53 |
| Negative cognitions about self, others and the world are characteristic of CPTSD, whereas they are not characteristic of EUPD. | 20 |
| Sense of self is stable and negative in CPTSD, whereas it can be unstable and fluctuating in EUPD. | 53 |
| Difficulties with mentalisation are not characteristic of CPTSD, whereas they can be characteristic of EUPD. | 33 |
| Emotional dysregulation is not characteristic of CPTSD, whereas it can be characteristic of EUPD. | 33 |
| Avoidance is linked to traumatic events in CPTSD, whereas it is linked to past negative experiences of relationships in EUPD. | 59 |
| Hypervigilance is indicative of CPTSD, whereas it is not indicative of EUPD. | 53 |
| Dissociation is characteristic of CPTSD, whereas it is not characteristic of EUPD. | 33 |
| Somatic symptoms are characteristic of CPTSD, whereas they are not characteristic of EUPD. | 33 |
| Lack of impulse control is not indicative of CPTSD, whereas it is often indicative of EUPD. | 40 |
| Repeated self-harm and/or suicidal behaviours are not characteristic of CPTSD, whereas they can be characteristic of EUPD. | 33 |
| Self-destructive behaviours (i.e., risky behaviour causing emotional or physical harm) are not common in CPTSD, whereas they are common in EUPD. | 33 |
| Psychological safety can reduce interpersonal difficulties in CPTSD, whereas interpersonal difficulties can remain present even if the person has a sense of psychological safety in EUPD. | 59 |
| Relationships are persistently negative or avoided in CPTSD, whereas they can fluctuate between extremes in EUPD. | 71 |
| Relating to others usually causes anxiety and so is often avoided in CPTSD, whereas others are experienced either positively or negatively at any one time in EUPD. | 76 |
| **Considerations for autism and CPTSD co-occurrence** |  |
| Autism is a risk factor for developing CPTSD (e.g., social and communication difficulties can increase the risk for vulnerability or maltreatment or traumatic events). | 76 |
| Autism-related difficulties overshadow CPTSD-related difficulties. | 47 |
| CPTSD-related difficulties overshadow autism-related difficulties. | 33 |
| **Considerations for autism and EUPD co-occurrence** |  |
| Autism-related difficulties overshadow EUPD-related difficulties. | 33 |
| EUPD-related difficulties overshadow autism-related difficulties. | 42 |
| Diagnosis of co-occurring autism and EUPD is complex to make as people age due to multiple factors (e.g., lack of developmental history, conflicting reports and opinions, lack of clarity about earlier diagnoses). | 69 |
| Autism and EUPD are not mutually exclusive and can co-occur. | 69 |
| **Considerations for CPTSD and EUPD co-occurrence** |  |
| In the presence of a history of traumatic events, CPTSD is a better explanation of difficulties found in adults diagnosed with EUPD. | 47 |
| Co-occurring EUPD and PTSD and sole CPTSD are each more common than co-occurring EUPD and CPTSD. | 20 |
| **Assessment methods for differential diagnosis of autism and CPTSD** |  |
| Dissociation measures (e.g., DES). | 36 |
| Theory of mind measures (e.g., RMET). | 36 |
| Mental state examination. | 36 |
| Personality traits measures (e.g., MMPI, SCID-PD). | 14 |
| Differentiating measures (e.g., Coventry Grid). | 36 |
| Executive function measures (e.g., ESQ). | 21 |
| Functional analysis of behaviour. | 67 |
| **Assessment methods for differential diagnosis of autism and EUPD** |  |
| Referring to diagnostic criteria (e.g., DSM, ICD). | 75 |
| Personality traits measures (e.g., IPDE, LPFS, MCMI, MMPI, PAI, PICD, SAPAS, SASPD, SCID-PD, STIPO). | 58 |
| EUPD measures (e.g., MSI-BPD). | 42 |
| Theory of mind measures (e.g., RMET). | 42 |
| Social functioning measures (e.g., SFQ). | 33 |
| Functional analysis of behaviour. | 58 |
| Cognitive assessments (e.g., WAIS). | 25 |
| Differentiating measures (e.g., Coventry Grid). | 42 |
| Observation of adults in natural settings (e.g., social contexts). | 69 |
| **Assessment methods for differential diagnosis of CPTSD and EUPD** |  |
| Referring to guidance (e.g., Cloitre et al., 2014, Felding et al., 2021). | 71 |
| Traumatic event exposure and trauma symptoms measures (e.g., ACE Questionnaire, CAPS, IES, ITI, PCL, PSSI SIDES, TRS). | 71 |
| Personality traits measures (e.g., IPDE, LPFS, MCMI, MMPI, PAI, PICD, SAPAS, SASPD, SCID-PD, STIPO). | 59 |
| EUPD measures (e.g., MSI-BPD). | 57 |
| Mental state examination. | 41 |
| Social functioning measures (e.g., SFQ). | 50 |
| **Difficulties in distinguishing autism and CPTSD** |  |
| Adults can self-identify with the autism diagnosis and may give expected answers to fulfil diagnostic criteria. | 67 |
| Cultural understanding of the aetiology of autism results in finding CPTSD less stigmatising than autism. | 57 |
| Clinicians are unfamiliar with CPTSD as it is a new diagnosis. | 62 |
| Decisions regarding accurate diagnosis can be based on access to treatment. | 57 |
| **Suggestions for improved distinction between autism and CPTSD** |  |
| Involvement of the multi-disciplinary team in all assessments. | 76 |
| Use of the Coventry Grid. | 33 |
| Inclusion of autism measures in all CPTSD assessments. | 53 |
| **Difficulties in distinguishing autism and EUPD** |  |
| Adults self-identify with the autism diagnosis and give expected answers to fulfil diagnostic criteria. | 58 |
| Females are assumed to have EUPD, and autism is overlooked. | 75 |
| Females are assumed to have atypical autism and EUPD is overlooked. | 19 |
| Childhood autism traits reported during a developmental history may be less pronounced in autistic females and autism is overlooked or misdiagnosed as EUPD. | 75 |
| Adults of lower socio-economic status are assumed to have EUPD, and autism is overlooked. | 69 |
| Access to assessment is unequal for those who experience marginalisation which may cause an escalation in presentation. | 69 |
| Differential diagnosis decision depends on access to appropriate services following diagnosis. | 63 |
| **Suggestions for improved distinction between autism and EUPD** |  |
| Involvement of the multi-disciplinary team in all assessments. | 58 |
| Improved autism diagnostic criteria (e.g., gender differences). | 58 |
| Inclusion of autism measures all in all EUPD assessments. | 50 |
| **Difficulties in distinguishing CPTSD and EUPD** |  |
| Adults self-identify with the CPTSD diagnosis and give expected answers to fulfil diagnostic criteria. | 53 |
| Females are assumed to have EUPD and CPTSD is overlooked. | 65 |
| If risk behaviours are present, EUPD is favoured by clinicians over CPTSD. | 65 |
| Adults with EUPD may have a significant trauma history, leading to misattribution and misdiagnosis of difficulties to CPTSD. | 53 |
| Differential diagnosis decision depends on access to appropriate services following diagnosis. | 47 |
| **Suggestions for improved distinction between CPTSD and EUPD** |  |
| * |  |

Items that received <80% of professionals’ agreement in the last round they were issued in. * No statements in this section.

1. Definition of acronyms used within consensus statements and the manuscript

- **AAA**: Adult Asperger Assessment
- **AACAP**: American Academy of Child and Adolescent Psychiatry
- **ACE** **Questionnaire**: Adverse Childhood Experiences Questionnaire
- **ADI**: Autism Diagnostic Interview
- **ADOS**: Autism Diagnostic Observation Schedule
- **AQ**: Autism Spectrum Quotient
- **Bar-On EQ**: Bar-On Emotional Quotient Inventory: Youth Version
- **BASC**: Behaviour Assessment System for Children
- **BRIEF**: Behavior Rating Inventory of Executive Function
- **BrownADDScales**: Brown Attention-Deficit Disorder Scales
- **CAI**: Child Attachment Interview
- **CAPS**: Clinician-Administered PTSD Scale
- **CARS**: Childhood Autism Rating Scale
- **CATI**: Comprehensive Autism Trait Inventory
- **CAT-Q**: Camouflaging Autistic Traits Questionnaire
- **CATS**: Child and Adolescent Trauma Screen
- **CPSS**: Child PTSD Symptom Scale
- **CPTSD**: Complex Post-Traumatic Stress Disorder
- **CRIES**: Child Revised Impact of Events Scale
- **DAI**: Disturbances of Attachment Interview
- **DAS**: Differential Ability Scales
- **DES**: Dissociative Experiences Scale
- **DISCO**: Diagnostic Interview for Social and Communication Disorders
- **DSED**: Disinhibited Social Engagement Disorder
- **DSM**: Diagnostic and Statistical Manual of Mental Disorders
- **DSO**: Disturbances in Self-Organisation
- **EQ**: Empathy Quotient
- **ESQ**: Executive Skills Questionnaire
- **EUPD**: Emotionally Unstable Personality Disorder
- **ICD:** International Classification of Diseases
- **IES**: Impact of Event Scale
- **IPDE**: International Personality Disorder Examination
- **ITI**: International Trauma Interview
- **ITQ**: International Trauma Questionnaire
- **LPFS**: Level of Personality Functioning Scale
- **MCMI**: Millon Clinical Multiaxial Inventory
- **MIDGAS**: Monteiro Interview Guidelines for Diagnosing the Autism Spectrum
- **MMPI**: Minnesota Multiphasic Personality Inventory
- **MSI**-**BPD**: McLean Screening Instrument for BPD
- **PAI**: Personality Assessment Inventory
- **PCL**: PTSD Checklist
- **PEDS**: Parents' Evaluation of Developmental Status
- **PICD**: Personality Inventory for ICD-11
- **PSSI**: PTSD Symptom Scale Interview
- **PTSD**: Post-Traumatic Stress Disorder
- **RAADS**: Ritvo Autism Asperger Diagnostic Scale
- **RAD**: Reactive Attachment Disorder
- **RMET**: Reading the Mind in the Eyes Task
- **RSCA**: Resiliency Scale for Children and Adolescents
- **SAPAS**: Structured Assessment of Personality Abbreviated Scale
- **SASPD**: Standardised Assessment of Severity of Personality Disorder
- **SAT**: Separation Anxiety Test
- **SCID-PD**: Structured Clinical Interview for DSM-5 Personality Disorders
- **SCQ**: Social Communication Questionnaire
- **SDQ**: Strengths and Difficulties Questionnaire
- **SFQ**: Social Functioning Questionnaire
- **SIDES**: Structured Interview for Disorders of Extreme Stress
- **SSAP**: Story Stem Assessment Profile
- **STIPO**: Structured Interview of Personality Organization
- **TRS**: Trauma Recovery Scale
- **TSCC**: Trauma Symptom Checklist for Children
- **TSCYC**: Trauma Symptom Checklist for Young Children
- **VABS**: Vineland Adaptive Behavior Scales
- **WAIS**: Wechsler Adult Intelligence Scale
- **WISC**: Wechsler Intelligence Scale for Children
